# Synopsis and meta-analysis of genetic association studies in osteoporosis for the focal adhesion family genes: the CUMAGAS-OSTEOporosis information system

**DOI:** 10.1186/1741-7015-9-9

**Published:** 2011-01-26

**Authors:** Elias Zintzaras, Chrysoula Doxani, Theocharis Koufakis, Alkibiadis Kastanis, Paraskevi Rodopoulou, Theofilos Karachalios

**Affiliations:** 1 Department of Biomathematics, University of Thessaly School of Medicine, Larissa, Greece; 2 Center for Clinical Evidence Synthesis, Institute for Clinical Research and Health Policy Studies, Tufts Medical Center, Tufts University School of Medicine, Boston, MA, USA; 3 Department of Orthopedics, University of Thessaly School of Medicine, Larissa, Greece

## Abstract

**Background:**

Focal adhesion (FA) family genes have been studied as candidate genes for osteoporosis, but the results of genetic association studies (GASs) are controversial. To clarify these data, a systematic assessment of GASs for FA genes in osteoporosis was conducted.

**Methods:**

We developed Cumulative Meta-Analysis of GAS-OSTEOporosis (CUMAGAS-OSTEOporosis), a web-based information system that allows the retrieval, analysis and meta-analysis (for allele contrast, recessive, dominant, additive and codominant models) of data from GASs on osteoporosis with the capability of update. GASs were identified by searching the PubMed and HuGE PubLit databases.

**Results:**

Data from 72 studies involving 13 variants of 6 genes were analyzed and catalogued in CUMAGAS-OSTEOporosis. Twenty-two studies produced significant associations with osteoporosis risk under any genetic model. All studies were underpowered (<50%). In four studies, the controls deviated from the Hardy-Weinberg equilibrium. Eight variants were chosen for meta-analysis, and significance was shown for the variants collagen, type I, α_1 _(*COL1A1*) G2046T (all genetic models), *COL1A1 *G-1997T (allele contrast and dominant model) and integrin β-chain β_3 _(*ITGB3*) T176C (recessive and additive models). In *COL1A1 *G2046T, subgroup analysis has shown significant associations for Caucasians, adults, females, males and postmenopausal women. A differential magnitude of effect in large versus small studies (that is, indication of publication bias) was detected for the variant *COL1A1 *G2046T.

**Conclusion:**

There is evidence of an implication of FA family genes in osteoporosis. CUMAGAS-OSTEOporosis could be a useful tool for current genomic epidemiology research in the field of osteoporosis.

## Background

Osteoporosis is a common skeletal disease characterized by generalized reduction in bone mineral density and microarchitectural deterioration of bone tissue, leading to impaired skeletal strength and increased susceptibility to fracture [1]. Genetic factors have long been recognized as playing an important role in osteoporosis [2]. Major efforts are currently underway to identify the specific genes and allelic variants predisposing patients to this disease. The identification of genes is achieved by conducting hypothesis-free, genome-wide association studies (GWASs) and candidate gene association studies (GASs) [3]. Candidate genes are typically chosen on the basis of having biological effects on bone metabolism or bone cell activity and whether they contribute to the risk of osteoporosis [3].

The focal adhesion (FA) gene family has emerged as a logical candidate for osteoporosis. Focal adhesions are specialized structures at the cellular-extracellular matrix contact points, where bundles of actin filaments are anchored to transmembrane receptors of the integrin family through a multimolecular complex of junctional plaque proteins. Some of the constituents of FA genes participate in the structural link between membrane receptors and the actin cytoskeleton, while others are signaling molecules [4,5]. Although there are a number of genes constituting the pathway, only a small number of variants of these genes have been studied in GASs of osteoporosis. More recently, haplotype-based approaches and genome-wide genotyping platforms have enabled more comprehensive capture of genetic variation in these genes [6]. The most studied gene is the collagen, type I, α_1 _(*COL1A1*) gene, especially its variant G2046T. Other genes studied in the FA family include the genes insulin-like growth factor I (*IGF-I*), integrin β-chain β_3 _(*ITGB3*), α-actinin-3 (*ACTN3*), *COL1A2 *and type 1 insulin-like growth factor receptor (*IGF-IR*). However, the results of the GASs involving genes of the FA pathway and osteoporosis are controversial and inconclusive, possibly because of methodological limitations, including inadequate sample size, patient selection, ethnicity of the populations studied and lack of adjustments for confounders [7].

To explore the involvement of FA family gene polymorphisms in osteoporosis susceptibility, we systematically searched for all available GASs of FA family genes and osteoporosis (as a binary phenotype) and created the Cumulative Meta-Analysis of Genetic Association Studies-OSTEOporosis (CUMAGAS-OSTEOporosis) information system. Then we catalogued all retrieved articles and estimated the risk effects of all individually investigated variants. Finally, the available data were synthesized using meta-analysis techniques to increase the power for detecting significant results and to decrease the uncertainty of the estimated genetic risks [8].

## Methods

### Information system

CUMAGAS-OSTEOporosis is a web-based database and an information system for cumulative meta-analysis of GASs [[9]; see also [10,11]]. CUMAGAS-OSTEOporosis performs meta-analysis for all genetic models (allele contrast, dominant, recessive, additive and codominant) and provides data on various covariates. Currently, CUMAGAS-OSTEOporosis operates for binary phenotypes (that is, osteoporosis: yes or no), but our study group is expanding the system to analyze continuous phenotype (bone mineral density).

CUMAGAS-OSTEOporosis is a dynamic system, since it has the capacity of continuous updating. Authors of published and unpublished studies may contribute their data by entering their studies' data into a prespecified data entry form (CUMAGAS-FORM) [9]. Furthermore, authors may correct previously stored data or notify for missed studies by contacting the CUMAGAS investigators (cumagas@med.uth.gr).

### Selection of Studies

All studies published before June 2010 were identified by conducting extended computer-based searches of the PubMed and HuGE PubLit databases. The search criteria in the PubMed database included a combination of the following terms: Focal adhesion, *ACP1, ACTN3, ADRB1, AKT1, COL1A1, COL1A2, COMP, CREBBP, CSNK1D, CTNNA3, CTNNB1, DRD2, FGFR1, GRB2, GRB2, IGF-I, IGF-IR*, *ITGA1, ITGA2, ITGA2B, ITGB3, KDR, PARD6A, PDGFRA, PDGFRB, PIK3CA, PRKACB, PTPN1, SMAD2, SMAD3, SMAD4, SPP1, TCF7, TGFBR1, TGFBR2, VEGFA, VWF*, osteoporosis, gene, polymorphism, allele and variant. The bibliographies in the articles that these searches identified were used to find further references. The HuGE PubLit database [12] was searched for the disease term "osteoporosis" and for the gene terms listed above.

The eligible studies fulfilled the following inclusion criteria: (1) inclusion of cases with clinically diagnosed osteoporosis and controls free of osteoporosis, (2) information provided on genotype frequency or risk estimates (only studies that reported a particular variant were considered, and missing data were not imputed), (3) use of DNA-based analytical methods for genotyping and (4) studies of humans. Studies investigating disease progression, severity, phenotype modification, response to treatment or survival were excluded from our study. Case reports, editorials, review articles and non-English-language articles were also excluded. Finally, family-based studies were excluded because of different design settings. Abstracts of retrieved studies were independently read by two investigators (CD and EZ) to assess their appropriateness for this study. Full-text articles of the studies were evaluated (by CD and EZ) according to the inclusion criteria. The results were compared, and disagreements were resolved by consensus.

Published GWASs of osteoporosis cited in the HuGE PubLit database [12] and the National Human Genome Research Institute Catalog of Published Genome-Wide Association Studies [13] were screened for variants of the FA gene family. Open access databases for GWASs [14] were also searched. The variants tested in candidate gene studies were examined regardless of whether they had been included or tagged by proxy variants in the genotyping platforms used in the GWASs of osteoporosis [15].

### Data abstraction

From each article, the following information was extracted: first author, year of publication, ethnicity of the study population, study design, demographics and number of cases and controls for each genotype and effect size. The frequencies of the alleles and the genotypic distributions were extracted or calculated for both the cases and the controls. The reference single nucleotide polymorphism identification numbers (rs numbers), the chromosomal gene position and the nucleotide base changes for all genetic variants were identified by performing extended searches of bioinformatics databases [12,14-16].

### Data analysis and synthesis

Prior to meta-analysis, the risk effect of gene variants for the allele contrast and the dominant models were evaluated separately for each study. All associations were indicated as odds ratios (ORs) with the corresponding 95% confidence intervals (95% CIs). In the meta-analysis, the heterogeneity between studies was tested using the Q-statistic [17,18], and it was quantified with the *I*^2 ^metric [8]. Heterogeneity was considered significant when *P*_Q _< 0.10 (*P*_*Q *_is the *P*-value for Q-statistic). The pooled OR was estimated using the random effects (RE) model [[19]; see also [8,20]]. The RE model was chosen because it is more conservative than the alternative fixed effects model, which does not consider heterogeneity. The differential magnitude of effect in large versus small studies (that is, indication of publication bias) was tested using a modified linear regression test for funnel plot asymmetry proposed by Harbord *et al*. [21]. This effect was considered significant when the *P*-value for Harbord's test was *P*_H_<0.05. The meta-analysis consisted of the main (that is, overall) analysis, which included all available data, as well as subgroup analyses by ethnicity, gender, age, menopausal status and sensitivity analysis which examined the effect of excluding specific studies [8,20].

The distribution of each variant in the control group was tested for the Hardy-Weinberg equilibrium (HWE) [22]. HWE indicates possible genotyping errors and/or population stratification [8]. Sensitivity analysis was carried out for the studies that deviated from HWE and the studies for which HWE could not be tested (that is, the pooled OR was calculated after excluding these studies). The power of each study for the allele contrast was calculated assuming a 20% alteration in effect size (that is, modest effect), a significance level of 0.05 and a disease allele frequency equal to the one of the study population [11]. Analyses were performed using the CUMAGAS-OSTEOporosis database [9] and Compaq Visual Fortran 90 software (Compaq Computer Corporation, Houston, Texas, US) with the International Mathematics and Statistics Library (Visual Nuemerics Inc, Houston, Texas, US).

## Results

### Eligible articles

**Figure 1:**
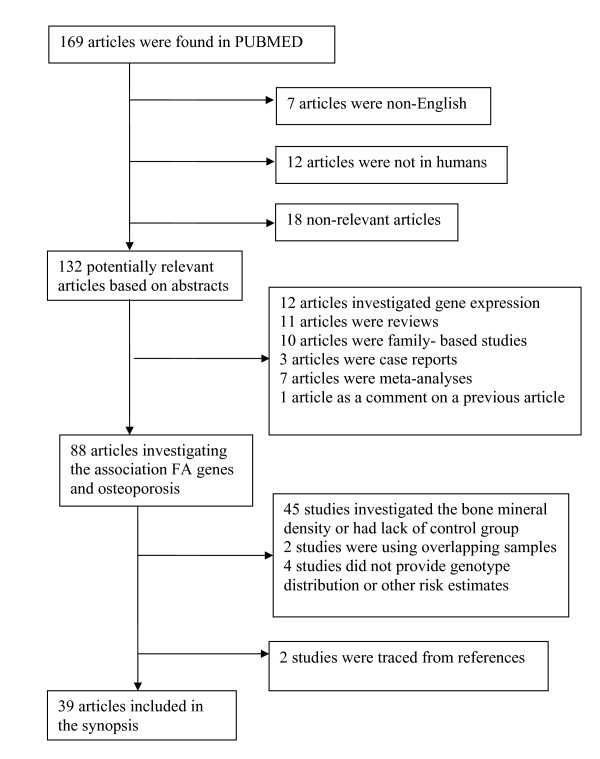
**Flowchart of retrieved studies and studies excluded, with specification of reasons**.

The literature review identified 169 titles in the PubMed and HuGELit databases that met the search criteria. The search in HuGE PubLit and the databases for GWASs traced articles already identified by PubMed. After abstract selection, 132 articles remained. When an article provided data for different populations, then each population was considered as a different study [23-27]. Thirty-nine articles consisting of 72 studies that investigated the association between genetic variants of the FA family genes and osteoporosis fulfilled the inclusion criteria. Figure 1 presents a flowchart of retrieved studies and studies that were excluded, with specification of the reasons for inclusion or exclusion (a list of the excluded studies is provided in Additional file 1). Overall, 6 genes and 13 distinct variants of these genes investigated in the 72 gene-disease association studies were identified. The studies were published between 1996 and 2010.

### Studies' characteristics and association results

The characteristics of each study and the association results of variants are shown in Additional file 2. In GWASs, none of the variants of the FA gene family were reported as significant, nor were the variants examined in the meta-analyses captured by the genotyping platforms used in the GWASs [28,29].

Studies were conducted in various populations of different racial descent: Sixty-six studies involved solely Caucasians, two studies recruited East Asians, three studies involved Turks and one study involved a population of Mexican origin. Twenty-three studies involved only postmenopausal women. Twelve studies provided data for men and women. One study involved children.

The distribution of genotypes in the control group deviated from HWE in 4 studies, and in 20 studies the HWE deviation could not be tested. In all studies, the statistical power for detecting a significant risk effect was lower than 50%. In total, 22 studies produced significant associations with osteoporosis risk under any genetic model. The significant associations concerned the variants *COL1A1 *G2046T, *COL1A1 Rsa*I intron 5, *COL1A2 Pvu*II and *ITGB3 *T176C.

### Meta-analysis results

In total, eight variants were investigated in two or more studies, and their results were meta-analyzed: *COL1A1 *G2046T (Sp1 SS/ss, rs1800012), *COL1A1 *G-1997T (rs1107946), *COL1A1 *-1663T ins/del (rs2412298), *COL1A1 Msp*I 26 kb upstream, *COL1A1 Rsa*I intron 5, *COL1A1 Mn*II exon 52, *IGF-I *192 bp CA and *ITGB3 *T176C (rs5920). Table 1 shows the meta-analysis results for the association between the different variants and the risk of developing osteoporosis. Significant results are shown for the variants *COL1A1 *G2046T, *COL1A1 *G-1997T and *ITGB3 *T176C. Additional file 3 shows the associations of the individual studies and the meta-analysis results for the dominant model of the variant *COL1A1 *G2046T.

**Table 1:** Meta-analysis results, odds ratios with corresponding 95% confidence intervals, heterogeneity metrics (*P*_Q_, *I*^2^) and significance of the differential magnitude of effect in large versus small studies (*P*_H_) for allele contrast and dominant and recessive models^a^

Gene	Study type	Population	Studies (n)	Cases/Controls (n)	OR (95% CI)	***I***^**2 **^**(%) (*P***_**Q **_**value)**	***P***_**H**_
*COL1A1 *G2046T	Allele contrast	All	33	7,308/10,128	1.65 (1.39-1.94)	69 (<0.01)	0.01
		All in HWE	31	6,660/8,968	1.68 (1.41-2.01)	70 (<0.01)	0.01
		Caucasians	28	6,896/9,645	1.47 (1.29-1.68)	50 (<0.01)	<0.01
		Adults	32	7,234/9,944	1.54 (1.33-1.78)	57 (<0.01)	0.01
		Female	21	5,942/8,104	1.37 (1.20-1.57)	40 (0.03)	0.05
		Postmenopausal	14	4,185/5,722	1.34 (1.14-1.58)	41 (0.06)	0.53
		Male	5	706/1,298	1.85 (1.09-3.14)	55 (0.06)	0.81
	Recessive model	All	33	3,661/5,071	2.37 (1.78-3.16)	16 (0.21)	0.26
		All in HWE	31	3,337/4,491	2.37 (1.73-3.25)	20 (0.16)	0.16
		Caucasians	28	3,592/5,021	2.29 (1.76-3.00)	2 (0.43)	0.08
		Adults	32	3,661/5,071	2.24 (1.75-2.88)	0 (0.48)	0.18
		Female	21	3,300/4,373	2.00 (1.52-2.63)	0 (0.54)	0.65
		Postmenopausal	14	2,496/3,261	1.80 (1.29-2.50)	2 (0.43)	0.85
		Male	5	757/1,049	5.47 (2.31-12.95)	0 (0.57)	0.11
	Dominant model	All	35	3,928/5,346	1.60 (1.34-1.91)	64 (<0.01)	0.01
		All in HWE	31	3,331/4,485	1.67 (1.37-2.03)	65 (<0.01)	0.03
		Caucasians	30	3,722/5,104	1.43 (1.23-1.65)	46 (<0.01)	<0.01
		Adults	34	3,891/5,254	1.53 (1.30-1.81)	58 (<0.01)	0.02
		Female	23	3,245/4,334	1.37 (1.18-1.58)	36 (0.05)	0.02
		Postmenopausal	15	2,311/3,013	1.33 (1.12-1.58)	33 (0.10)	0.41
		Male	5	354/650	1.54 (0.81-2.93)	54 (0.07)	0.98
	Additive model	All	33	2,487/3,658	2.64 (1.93-3.60)	24 (0.11)	0.13
		All in HWE	31	2,267/3,275	2.68 (1.90-3.78)	28 (0.08)	0.07
		Caucasians	28	2,447/3,626	2.46 (1.86-3.26)	9 (0.33)	0.03
		Adults	32	2,487/3,658	2.57 (1.94-3.41)	11 (0.29)	0.03
		Female	21	2,242/3,159	2.14 (1.59-2.89)	8 (0.35)	0.31
		Postmenopausal	14	1,694/2,399	1.94 (1.31-2.86)	20 (0.24)	0.55
		Male	5	547/843	5.18 (2.16-12.42)	0 (0.58)	0.20
	Codominant	All	49	9,722/22,924	1.17 (1.04-1.31)	69 (<0.01)	0.13
		All in HWE	31	3,331/4,485	1.33 (1.11-1.60)	57 (<0.01)	0.31
		Caucasians	44	9,516/22,682	1.12 (1.01-1.26)	68 (<0.01)	0.14
		Adults	48	9,685/22,832	1.17 (1.04-1.31)	69 (<0.01)	0.13
		Female	30	7,497/16,464	1.12 (0.99-1.26)	62 (<0.01)	0.38
		Postmenopausal	15	3,290/6,530	1.16 (0.98-1.37)	36 (0.08)	0.26
		Male	12	1,896/6,098	1.07 (0.80-1.44)	78 (<0.01)	0.29
*COL1A1 *G-1997T	Allele contrast	All	3	778/852	1.38 (1.05-1.81)	0 (0.94)	0.11
	Recessive model	All	3	389/426	1.66 (0.67-4.11)	0 (0.70)	0.95
	Dominant model	All	3	389/426	1.42 (1.04-1.93)	0 (0.88)	0.53
	Additive model	All	3	279/330	1.08 (0.72-4.49)	0 (0.73)	0.93
	Codominant	All	3	389/426	1.36 (0.99-1.87)	0 (0.73)	0.71
*COL1A1 *-1663T ins/del	Allele contrast	All	3	776/850	1.03 (0.81-1.33)	0 (0.73)	0.14
	Recessive model	All	3	388/425	1.81 (0.87-3.71)	0 (0.78)	0.11
	Dominant model	All	3	388/425	0.94 (0.70-1.26)	0 (0.49)	0.09
	Additive model	All	3	283/295	1.74 (0.84-3.58)	0 (0.86)	0.20
	Codominant	All	3	388/425	0.82 (0.58-1.15)	12 (0.32)	0.06
*COL1A1 Msp*I 26 kb upstream (mt^-^/wt^+^)	Allele contrast	All	2	318/312	1.33 (0.95-1.86)	NA (0.86)	NA
	Recessive model	All	2	159/156	1.44 (0.69-2.98)	NA (0.51)	NA
	Dominant model	All	2	159/156	1.43 (0.91-2.23)	NA (0.92)	NA
	Additive model	All	2	88/94	1.66 (0.78-3.56)	NA (0.50)	NA
	Codominant	All	2	159/156	1.25 (0.79-1.97)	NA (0.87)	NA
*COL1A1 Rsa*I intron 5 (mt^-^/wt^+^)	Allele contrast	All	2	318/312	1.03 (0.37-2.91)	NA (0.01)	NA
	Recessive model	All	2	159/156	0.85 (0.27-2.66)	NA (0.47)	NA
	Dominant model	All	2	159/156	1.09 (0.29-4.19)	NA (<0.01)	NA
	Additive model	All	2	96/103	0.90 (0.23-3.42)	NA (0.25)	NA
	Codominant	All	2	159/156	1.13 (0.33-3.89)	NA (0.01)	NA
*COL1A1 Mn*II exon 52 (mt^-^/wt^+^)	Allele contrast	All	2	318/312	0.77 (0.55-1.07)	NA (0.41)	NA
	Recessive model	All	2	159/156	0.70 (0.18-2.75)	NA (0.07)	NA
	Dominant model	All	2	159/156	0.78 (0.50-1.22)	NA (0.84)	NA
	Additive model	All	2	96/93	0.63 (0.20-2.00)	NA (0.14)	NA
	Codominant	All	2	159/156	0.95 (0.47-1.93)	NA (0.12)	NA
*IGF-I *192 bp CA (mt^-^/wt^+^)	Dominant model	All	2	47/136	0.70 (0.15-3.35)	NA (0.03)	NA
*ITGB3 *T176C	Allele contrast	All	2	534/17,932	1.23 (0.87-1.76)	NA (0.11)	NA
	Recessive model	All	2	267/8,966	1.88 (1.06-3.33)	NA (0.76)	NA
	Dominant model	All	2	267/8,966	1.20 (0.76-1.90)	NA (0.09)	NA
	Additive model	All	2	192/6,517	1.93 (1.09-3.45)	NA (0.58)	NA
	Codominant	All	2	267/8,966	1.09 (0.68-1.72)	NA (0.10)	NA

^a^OR, odds ratio; 95% CI, 95% confidence interval; *I*^2^, quantification of heterogeneity; *P*_Q_, *P*-value for heterogeneity; *P*_H_, *P*-value for magnitude of effect in large versus small studies; *COL1A1*, collagen, type I, α_1_; NA, not applicable; HWE, Hardy-Weinberg equilibrium; mt, mutant type; wt, wild type; *IGF-I*, insulin-like growth factor I; *ITGB3*, integrin β-chain β_3_.

In particular, there was a significant overall association for the allele contrast of the variant *COL1A1 *G2046T (OR, 1.65; 95% CI, 1.39-1.94) with the heterogeneity between studies being significant (*I*^2 ^= 69%; *P*_Q _< 0.01). In subgroup analysis, a significant association was shown for Caucasians (OR, 1.60; 95% CI, 1.36-1.88), adults (OR, 1.48; 95% CI, 1.29-1.70), females (OR, 1.37; 95% CI, 1.20-1.57), males (OR, 1.85; 95% CI, 1.09-3.14) and postmenopausal women (OR, 1.34; 95% CI, 1.14-1.58). The recessive, dominant, additive and codominant models produced similar results (with the exception of subgroup analysis for males for the recessive and additive models). The sensitivity analysis for HWE did not alter the pattern of results.

Significant results were also shown for the variants *COL1A1 *G-1997T (allele contrast and dominant model) and *ITGB3 *T176C (recessive and additive models). However, these results were based on a small number of studies, and therefore safe conclusions could not be drawn. In the overall meta-analyses for the allele contrast, a differential magnitude of effect in large versus small studies was detected only for the variant *COL1A1 *G2046T (*P*_H _= 0.01).

## Discussion

In this project, the currently available data from GASs on human FA family genes in osteoporosis were catalogued. Then the data were synthesized, and the involvement of FA gene variants in disease susceptibility was assessed comprehensively. The eligible GASs were catalogued in a publicly available web-based database and information system called CUMAGAS-OSTEOporosis [9]. In total, eight variants were meta-analyzed. Significant results were shown for the variants *COL1A1 *G2046T, *COL1A1 *G-1997T and *ITGB3 *T176C. Type I collagen is the major protein constituent of bone and is therefore a strong and plausible candidate gene for osteoporosis. The *COL1A1 *G2046T polymorphism is a single base pair substitution (G → T) within the regulatory region of the *COL1A1 *gene [30]. The *COL1A1 *G-1997T polymorphism has been identified in the proximal promoter of *COL1A1 *at position -1997 and is in linkage disequilibrium with the *COL1A1 *G2046T polymorphism [31]. There is evidence that the promoter polymorphisms are functional and have effects on DNA binding and gene transcription [32], but it is unclear to what extent this polymorphism is associated with the biomechanical properties of bone or susceptibility to fracture. Taking into consideration the abundance of *COL1A1 *in the bones and the fact that these variants seem to play a major role in the function of the gene, the significant association found in our results may encourage intensive research in this area. The *ITGB3 *T176C polymorphism changes the conformational structure of the β_3_-subunit of integrin [33]. The integrin β_3_-subunit is known to play a key role in the resorptive function of osteoclasts, as shown *in vitro *and in transgenic animal studies [34]. Genetic variation in integrin β_3 _may influence bone remodeling and subsequent bone loss and risk of osteoporotic fractures.

CUMAGAS-OSTEOporosis is an evidence-based information system for systematically searching, reviewing and synthesizing data for GASs of osteoporosis, with the capacity for continuous updating. CUMAGAS is being expanded to additional complex diseases such chronic lymphocytic leukemia, peripheral arterial disease, hypertension and osteoarthritis [10,9,35,36]. CUMAGAS also has the capacity to incorporate data from GWASs subject to their public availability.

The GWASs of osteoporosis have not highlighted a significant role for the FA family of genes. However, the commercial genotyping platforms [15] may underrepresent the variants of the FA pathway and, of course, the variants included in the meta-analysis. In addition, the variants identified to date from the GWAS approach explain only a fraction of the disease heritability, and therefore the potential role for the variants of FA pathway may not be excluded [36]. Furthermore, the analyses of GWASs have missed associations of multilocus variants involved in pathways with pathophysiological relevance to disease mechanisms [3,36].

Most of the published GASs are underpowered for detecting the minor contribution of common alleles. For example, a sample size of more than 10,000 patients is needed to achieve >80% power to detect a significant OR between 1.1 and 1.5 (modest effect) for a polymorphic locus in association with a complex disease [37]. Meta-analysis is a tool that allows for analysis with the potential for higher power by pooling the results of multiple studies [8]. However, there is no formal, established methodology for calculating the power of a meta-analysis. In addition, power analysis may not be applicable in meta-analysis, since it is a retrospective, all-inclusive synthesis of published studies [8,38]. Nevertheless, type II errors are expected to be less common in a meta-analysis than in single studies [8,39]. Currently, no single institution alone is able to provide a sufficient number of patients, and therefore the creation of large databases from consortia where researchers share their data are required. However, this need for data sharing has been pointed out by previous initiatives [40,41].

Two problems in human genome epidemiology research are that negative studies are frequently unpublished and some studies do not provide extractable data, which lead to the well-known phenomenon of publication bias [42]. However, negative results should also have a venue for publication. Moreover, the inclusion of "negative" and unpublished data in meta-analyses of GASs as a means of reducing publication bias is commonly suggested and is believed to help in pointing out genetic effects [43]. Thus, the establishment of an electronic information system to aid in performing cumulative meta-analyses of (published and unpublished) GASs of osteoporosis and identifying significant genetic variants could be a valuable tool for ongoing research in the field. Furthermore, CUMAGAS-OSTEOporosis will support rapid progress in human genome epidemiology of osteoporosis by identifying valid and replicable associations and making the overall effect of each variant from published and unpublished studies rapidly available to researchers. Concerning the retrieval of unpublished studies, the authors of unpublished studies will be able to submit their data to the CUMAGAS-OSTEOporosis database. Since these data will not have undergone peer review, a sensitivity analysis may be carried out (that is, a meta-analysis that examines the effect of excluding these studies). Finally, CUMAGAS-OSTEOporosis is an open access system, and it may support efforts to prevent publication bias [44].

Publication bias was tested using the method proposed by Harbord *et al*. [21], which is a modification of Egger's test [45], and it is appropriate for small-study effects. The visual inspection of funnel plots was avoided, since their validity is questionable [46,47]. However, the statistical tests used to evaluate studies for publication bias actually compare the differential magnitude of effects in large versus small studies [8,48].

The significance of risk effects in the GASs was assessed using the OR metric for various genetic models (dominant, recessive, additive and codominant) by merging genotypes. These models are not independent, and there is no *a priori *biological justification for their choice. Consequently, the interpretation of the results can be problematic, especially when all genetic contrasts are significant as in the case of *COL1A1 *G2046T. In these cases, the introduction of the recently proposed generalized OR (OR_G_) as an overall genetic risk effect may be a remedy [42]. The OR_G _is a single statistic that summarizes the magnitude and significance of the association without considering the hash of possible contrasts, and thus the interpretation of the results is straightforward [49]. The meta-analysis methodology in CUMAGAS-OSTEOporosis is expanding to incorporate the OR_G _metric and the continuous phenotype of osteoporosis (that is, bone mineral density).

In the meta-analysis, various genetic contrasts in different populations were explored, since there is no biological justification for choosing a specific contrast [49]. Thus, adjustment for multiple testing is not strictly required for such an exploratory study [8,50]. In addition, the adjustment for multiple testing might not be necessary, since the data were synthesized with the objective of reducing the uncertainty of effect size, without a prespecified hypothesis [50-52]. Furthermore, an appropriate multiple test adjustment might be difficult because the investigated contrasts are not independent and there is no clear structure in the multiple tests [49,52]. Finally, an adjustment for multiple comparisons (for example, Bonferroni's correction) concerns a general null hypothesis that there is no association in all genetic contrasts simultaneously, which is not likely [53,54].

The phenotypic heterogeneity of osteoporosis in the included studies in our synopsis makes the effort to combine the findings of GASs meaningfully in the complex field of osteoporosis a difficult task. Thus, in the presence of the large heterogeneity, the results should be interpreted with caution. There may be fundamental differences in the nature of genetic susceptibility to osteoporosis between postmenopausal and premenopausal women and between the two sexes or even among patients of different ethnicities. Furthermore, environmental factors, particularly nutrients, have to be accurately evaluated together with complex genotyping, to weigh their importance in revealing functional variants with respect to specific genetic background [55,56]. Our analysis used the available study-level allele and genotype distributions, precluding adjusted analysis for potential gene-gene and gene-environment interactions, for which raw genotype data would be required. Failure to account for interactions may have reduced the power of our analysis but is unlikely to have inflated the number of positive results.

## Conclusion

There is evidence implicating the activity of the FA family of genes in osteoporosis. Future studies designed to investigate epistatic and gene-environment interactions may help in deriving more conclusive claims about the role of these genes in osteoporosis. The CUMAGAS-OSTEOporosis information system can be a useful resource for reviewing and interpreting the findings of the accumulating genomic epidemiologic research in osteoporosis.

## Competing interests

The authors declare that they have no competing interests.

## Authors' contributions

TK conceived the study. EZ and TK were the principal investigators. EZ, CD and TK retrieved and assessed the articles. EZ, AK and TM developed the algorithms and the information system. EZ and PR performed the analysis. EZ, CD and TK drafted the manuscript. All authors read and approved the final manuscript.

## Pre-publication history

The pre-publication history for this paper can be accessed here:

http://www.biomedcentral.com/1741-7015/9/9/prepub

## Supplementary Material

Additional file 1 Supplementary Table 1 Click here for file

Additional file 2 Supplementary Table 2 Click here for file

Additional file 3 Supplementary Figure 1 Click here for file
