# Supplementary material for: Synopsis and meta-analysis of genetic association studies in osteoporosis for the focal adhesion family genes: the CUMAGAS-OSTEOporosis information system
Source: BMC Med. 2011 Jan 26;9:9. doi: 10.1186/1741-7015-9-9 (PMC3040157; doi:10.1186/1741-7015-9-9)
Supplement: Additional file 1 — Supplementary Table 1 [file 1741-7015-9-9-S1.PDF]

Articles in English and humans excluded from the synopsis

12 articles were about the gene and gene expression [1-11, 163]
11 articles were reviews [12-22]
13 articles were for irrelevant phenotype [23-33, 166, 167]
10 articles were family- based studies [34- 43]
12 articles were not conducted in humans [44- 54, 164]
3 articles were case reports [55-57]
1 article as a comment on a previous article [58]
7 articles were not in English [59-65]
5 articles were meta analyses [66-70]
4 articles were for other genotype [71- 74]
1 article was irrelevant [75]
45 studies investigated the bone mineral density or had lack of control group [76-92, 94-121]
2 studies were using overlapping samples [124, 125]
4 studies did not provide genotype distribution or other statistics [126-129]
2 Were meta-analyses of GWAS [130, 131]
39 articles used [93, 94, 122, 123, 124, 132- 162, 165, 166, 169]

REFERENCES

1. Dalle Carbonare L, Valenti MT, Zanatta M, Donatelli L, Lo Cascio V. Circulating mesenchymal stem cells with abnormal osteogenic differentiation in patients with osteoporosis. *Arthritis Rheum. 2009 Nov;60(11):3356-65.*
2. Balla B, Kósa JP, Kiss J, Borsy A, Podani J, Takács I, Lazáry A, Nagy Z, Bácsi K, Speer G, Orosz L, Lakatos P. Different gene expression patterns in the bone tissue of aging postmenopausal osteoporotic and non-osteoporotic women. *Calcif Tissue Int. 2008 Jan;82(1):12-26. Epub 2007 Dec 12.*
3. García-Giralt N, Enjuanes A, Bustamante M, Mellibovsky L, Nogués X, Carreras R, Díez-Pérez A, Grinberg D, Balcells S.In vitro functional assay of alleles and haplotypes of two COL1A1-promoter SNPs. *Bone. 2005 May;36(5):902-8. Epub 2005 Apr 7*
4. Ozawa S, Ogawa T, Iida K, Sukotjo C, Hasegawa H, Nishimura RD, Nishimura I. Ovariectomy hinders the early stage of bone-implant integration: histomorphometric, biomechanical, and molecular analyses. *Bone. 2002 Jan;30(1):137-43.*
5. Dalgleish R. The human type I collagen mutation database. *Nucleic Acids Res. 1997 Jan 1;25(1):181-7.*
6. Hopwood B, Tsykin A, Findlay DM, Fazzalari NL.Gene expression profile of the bone microenvironment in human fragility fracture bone. Bone. 2009 Jan;44(1):87-101
7. Effenberger KE, Johnsen SA, Monroe DG, Spelsberg TC, Westendorf JJ. Regulation of osteoblastic phenotype and gene expression by hop-derived phytoestrogens. J Steroid Biochem Mol Biol. 2005 Sep;96(5):387-99.
8. Sakamoto W, Isomura H, Fujie K, Deyama Y, Kato A, Nishihira J, Izumi H.Homocysteine attenuates the expression of osteocalcin but enhances osteopontin in MC3T3-E1 preosteoblastic cells. Biochim Biophys Acta. 2005 Apr 15;1740(1):12-6.
9. Stevens DA, Harvey CB, Scott AJ, O'Shea PJ, Barnard JC, Williams AJ, Brady G, Samarut J, Chassande O, Williams GR.Thyroid hormone activates fibroblast growth factor receptor-1 in bone. Mol Endocrinol. 2003 Sep;17(9):1751-66
10. Walker LM, Preston MR, Magnay JL, Thomas PB, El Haj AJ. Nicotinic regulation of c-fos and osteopontin expression in human-derived osteoblast-like cells and human trabecular bone organ culture. Bone. 2001 Jun;28(6):603-8.

11. Glass DA 2nd, Karsenty G. Molecular bases of the regulation of bone remodeling by the canonical Wnt signaling pathway. Curr Top Dev Biol. 2006;73:43-84.
12. Jaakkola E, Laine CM, Mäyränpää MK, Falck A, Ignatius J, Mäkitie O. Calvarial doughnut lesions and osteoporosis: a new three-generation family and review. *Am J Med Genet A. 2009 Nov;149A(11):2371-7.* Review.
13. Ferrari SL, Rizzoli R. Gene variants for osteoporosis and their pleiotropic effects in aging. *Mol Aspects Med. 2005 Jun;26(3):145-67.*
14. Ferrari S, Rizzoli R, Bonjour JP.Genetic aspects of osteoporosis. *Curr Opin Rheumatol. 1999 Jul;11(4):294-300.*
15. Kuivaniemi H, Tromp G, Prockop DJ.Mutations in collagen genes: causes of rare and some common diseases in humans. FASEB J. 1991 Apr;5(7):2052-60.
16. Haussler MR, Haussler CA, Whitfield GK, Hsieh JC, Thompson PD, Barthel TK, Bartik L, Egan JB, Wu Y, Kubicek JL, Lowmiller CL, Moffet EW, Forster RE, Jurutka PW. The nuclear vitamin D receptor controls the expression of genes encoding factors which feed the "Fountain of Youth" to mediate healthful aging. J Steroid Biochem Mol Biol. 2010 Mar 20.
17. Li WF, Hou SX, Yu B, Li MM, Férec C, Chen JM.Genetics of osteoporosis: accelerating pace in gene identification and validation. Hum Genet. 2010 Mar;127(3):249-85.
18. Cluett C, Melzer D. Human genetic variations: Beacons on the pathways to successful ageing. Mech Ageing Dev. 2009 Sep;130(9):553-63.
19. Hoeppner LH, Secreto FJ, Westendorf JJ.Wnt signaling as a therapeutic target for bone diseases. Expert Opin Ther Targets. 2009 Apr;13(4):485-96.
20. Tinkle BT, Wenstrup RJ. A genetic approach to fracture epidemiology in childhood. Am J Med Genet C Semin Med Genet. 2005 Nov 15;139C(1):38-54
21. Rawadi G, Roman-Roman S. Wnt signalling pathway: a new target for the treatment of osteoporosis. Expert Opin Ther Targets. 2005 Oct;9(5):1063-77.
22. Niu T, Rosen CJ. The insulin-like growth factor-I gene and osteoporosis: a critical appraisal. Gene. 2005 Nov 21;361:38-56. Epub 2005 Sep
23. Obafemi AA, Bulas DI, Troendle J, Marini JC. Popcorn calcification in osteogenesis imperfecta: incidence, progression, and molecular correlation. *Am J Med Genet A. 2008 Nov 1;146A(21):2725-32.*
24. Clayton AE, Mikulec AA, Mikulec KH, Merchant SN, McKenna MJ. Association between osteoporosis and otosclerosis in women. *J Laryngol Otol. 2004 Aug;118(8):617-21.*
25. McKenna MJ, Nguyen-Huynh AT, Kristiansen AG. Association of otosclerosis with Sp1 binding site polymorphism in COL1A1 gene: evidence for a shared genetic etiology with osteoporosis. *Otol Neurotol. 2004 Jul;25(4):447-50.*
26. Hampson G, Evans C, Petitt RJ, Evans WD, Woodhead SJ, Peters JR, Ralston SH. Bone mineral density, collagen type 1 alpha 1 genotypes and bone turnover in premenopausal women with diabetes mellitus. *Diabetologia. 1998 Nov;41(11):1314-20.*
27. Spotila LD, Colige A, Sereda L, Constantinou-Deltas CD, Whyte MP, Riggs BL, Shaker JL, Spector TD, Hume E, Olsen N, et al.Mutation analysis of coding sequences for type I procollagen in individuals with low bone density. J Bone Miner Res. 1994 Jun;9(6):923-32
28. Khoschnau S, Melhus H, Jacobson A, Rahme H, Bengtsson H, Ribom E, Grundberg E, Mallmin H, Michaëlsson K Type I Collagen {alpha}1 Sp1 Polymorphism and the Risk of Cruciate Ligament Ruptures or Shoulder Dislocations.
The American journal of sports medicine 2008 Jul .
29. Pawlikowska L, Hu D, Huntsman S, Sung A, Chu C, Chen J, Joyner AH, Schork NJ, Hsueh WC, Reiner AP, Psaty BM, Atzmon G, Barzilai N, Cummings SR, Browner WS, Kwok PY, Ziv E; Study of Osteoporotic Fractures. Association of

common genetic variation in the insulin/IGF1 signaling pathway with human longevity. Aging Cell. 2009 Aug;8(4):460-72.

30. Lindahl K, Rubin CJ, Brändström H, Karlsson MK, Holmberg A, Ohlsson C, Mellström D, Orwoll E, Mallmin H, Kindmark A, Ljunggren O. Heterozygosity for a coding SNP in COL1A2 confers a lower BMD and an increased stroke risk. Biochem Biophys Res Commun. 2009 Jul 10;384(4):501-5.
31. Faqeih E, Roughley P, Glorieux FH, Rauch F.Osteogenesis imperfecta type III with intracranial hemorrhage and brachydactyly associated with mutations in exon 49 of COL1A2. Am J Med Genet A. 2009 Mar;149A(3):461-5.
32. Hopwood B, Tsykin A, Findlay DM, Fazzalari NL. Microarray gene expression profiling of osteoarthritic bone suggests altered bone remodelling, WNT and transforming growth factor-beta/bone morphogenic protein signalling. Arthritis Res Ther. 2007;9(5):R100.
33. Taylor BC, Schreiner PJ, Doherty TM, Fornage M, Carr JJ, Sidney S. Matrix Gla protein and osteopontin genetic associations with coronary artery calcification and bone density: the CARDIA study. Hum Genet. 2005 May;116(6):525-8.
34. Xia XY, Cui YX, Huang YF, Pan LJ, Yang B, Wang HY, Li XJ, Shi YC, Lu HY, Zhou YC. A novel RNA-splicing mutation in COL1A1 gene causing osteogenesis imperfecta type I in a Chinese family. *Clin Chim Acta. 2008 Dec;398(1-2):148-51. Epub 2008 Aug 5.*
35. Kaufman JM, Ostertag A, Saint-Pierre A, Cohen-Solal M, Boland A, Van Pottelbergh I, Toye K, de Vernejoul MC, Martinez M. Genome-wide linkage screen of bone mineral density (BMD) in European pedigrees ascertained through a male relative with low BMD values: evidence for quantitative trait loci on 17q21-23, 11q12-13, 13q12-14, and 22q11. *J Clin Endocrinol Metab. 2008 Oct;93(10):3755-62. Epub 2008 Jul 29.*
36. Chen XD, Shen H, Lei SF, Li MX, Yang YJ, Deng HW. Exclusion mapping of chromosomes 1, 4, 6 and 14 with bone mineral density in 79 Caucasian pedigrees. *Bone. 2006 Mar;38(3):450-5. Epub 2005 Oct 24.*
37. Xu FH, Liu YJ, Deng H, Huang QY, Zhao LJ, Shen H, Liu YZ, Dvornyk V, Conway T, Li JL, Davies KM, Recker RR, Deng HW.A follow-up linkage study for bone size variation in an extended sample. *Bone. 2004 Sep;35(3):777-84*
38. Long JR, Liu PY, Lu Y, Dvornyk V, Xiong DH, Zhao LJ, Deng HW. Tests of linkage and/or association of TGF-beta1 and COL1A1 genes with bone mass. *Osteoporos Int. 2005 Jan;16(1):86-92. Epub 2004 May 26.*
39. Long JR, Liu PY, Lu Y, Xiong DH, Zhao LJ, Zhang YY, Elze L, Recker RR, Deng HW. Association between COL1A1 gene polymorphisms and bone size in Caucasians. *Eur J Hum Genet. 2004 May;12(5):383-8.*
40. Brown MA, Haughton MA, Grant SF, Gunnell AS, Henderson NK, Eisman JA.Genetic control of bone density and turnover: role of the collagen 1alpha1, estrogen receptor, and vitamin D receptor genes. *J Bone Miner Res. 2001 Apr;16(4):758-64.*
41. Hustmyer FG, Liu G, Johnston CC, Christian J, Peacock M. Polymorphism at an Sp1 binding site of COL1A1 and bone mineral density in premenopausal female twins and elderly fracture patients. *Osteoporos Int. 1999;9(4):346-50.*
42. Lau HH, Ng MY, Cheung WM, Paterson AD, Sham PC, Luk KD, Chan V, Kung AW Assessment of linkage and association of 13 genetic loci with bone mineral density.
Journal of bone and mineral metabolism 2006 24 (3): 226-34.
43. Gao G, Zhang ZL, He JW, Zhang H, Yue H, Hu WW, Gu JM, Fu WZ, Hu YQ, Li M, Liu YJ, Yu JB.No association of the polymorphisms of the frizzled-related protein gene with peak bone mineral density in Chinese nuclear families. BMC Med Genet. 2010 Jan 1;11:1.
44. Lisse TS, Thiele F, Fuchs H, Hans W, Przemeck GK, Abe K, Rathkolb B, Quintanilla-Martinez L, Hoelzlwimmer G, Helfrich M, Wolf E, Ralston SH,

Hrabé de Angelis M. ER stress-mediated apoptosis in a new mouse model of osteogenesis imperfecta. *PLoS Genet. 2008 Feb;4(2):e7.*

45. Ono N, Nakashima K, Schipani E, Hayata T, Ezura Y, Soma K, Kronenberg HM, Noda M. Constitutively active parathyroid hormone receptor signaling in cells in osteoblastic lineage suppresses mechanical unloading-induced bone resorption. *J Biol Chem. 2007 Aug 31;282(35):25509-16. Epub 2007 May 11.*
46. Forlino A, Porter FD, Lee EJ, Westphal H, Marini JC. Use of the Cre/lox recombination system to develop a non-lethal knock-in murine model for osteogenesis imperfecta with an alpha1(I) G349C substitution. Variability in phenotype in BrtlIV mice. *J Biol Chem. 1999 Dec 31;274(53):37923-31.*
47. Pereira RF, Hume EL, Halford KW, Prockop DJ. Bone fragility in transgenic mice expressing a mutated gene for type I procollagen (COL1A1) parallels the age-dependent phenotype of human osteogenesis imperfecta. *J Bone Miner Res. 1995 Dec;10(12):1837-43.*
48. Xiao Y, Cui J, Li YX, Shi YH, Le GW. Expression of genes associated with bone resorption is increased and bone formation is decreased in mice fed a high-fat diet. Lipids. 2010 Apr;45(4):345-55.
49. Tang DZ, Hou W, Zhou Q, Zhang M, Holz J, Sheu TJ, Li TF, Cheng SD, Shi Q, Harris SE, Chen D, Wang YJ.Osthole stimulates osteoblast differentiation and bone formation by activation of beta-catenin-BMP signaling. J Bone Miner Res. 2010 Jan 15.
50. Kansara M, Tsang M, Kodjabachian L, Sims NA, Trivett MK, Ehrich M, Dobrovic A, Slavin J, Choong PF, Simmons PJ, Dawid IB, Thomas DM. Wnt inhibitory factor 1 is epigenetically silenced in human osteosarcoma, and targeted disruption accelerates osteosarcomagenesis in mice. J Clin Invest. 2009 Apr;119(4):837-51.
51. Almeida M, Han L, Martin-Millan M, O'Brien CA, Manolagas SC. Oxidative stress antagonizes Wnt signaling in osteoblast precursors by diverting beta-catenin from T cell factor- to forkhead box O-mediated transcription. J Biol Chem. 2007 Sep 14;282(37):27298-305.
52. Hittmeier LJ, Grapes L, Lensing RL, Rothschild MF, Stahl CH. Genetic background influences metabolic response to dietary phosphorus restriction. J Nutr Biochem. 2006 Jun;17(6):385-95.
53. Verberckmoes SC, De Broe ME, D'Haese PC Dose-dependent effects of strontium on osteoblast function and mineralization.. Kidney Int. 2003 Aug;64(2):534-43.
54. O'Shea PJ, Harvey CB, Suzuki H, Kaneshige M, Kaneshige K, Cheng SY, Williams GR.A thyrotoxic skeletal phenotype of advanced bone formation in mice with resistance to thyroid hormone. Mol Endocrinol. 2003 Jul;17(7):1410-24.
55. Spotila LD, Constantinou CD, Sereda L, Ganguly A, Riggs BL, Prockop DJ. Mutation in a gene for type I procollagen (COL1A2) in a woman with postmenopausal osteoporosis: evidence for phenotypic and genotypic overlap with mild osteogenesis imperfecta. *Proc Natl Acad Sci U S A. 1991 Jun 15;88(12):5423-7.*
56. Barbirato C, Almeida MG, Milanez M, Sipolatti V, Rebouças MR, Akel AN Jr, Nunes VR, Perrone AM, Zatz M, Louro ID, Paula F.A novel COL1A1 gene-splicing mutation (c.1875+1G>C) in a Brazilian patient with osteogenesis imperfecta. Genet Mol Res. 2009 Feb 17;8(1):173-8.
57. Shapiro JR, Stover ML, Burn VE, McKinstry MB, Burshell AL, Chipman SD, Rowe DW. An osteopenic nonfracture syndrome with features of mild osteogenesis imperfecta associated with the substitution of a cysteine for glycine at triple helix position 43 in the pro alpha 1(I) chain of type I collagen. J Clin Invest. 1992 Feb;89(2):567-73.

58. Zajícková K, Zofková I, Hill M. Is the Sp1 polymorphism in the COL1A1 gene a risk factor for postmenopausal osteoporosis? Comment on the article by Keen et al. *Arthritis Rheum. 2002 Oct;46(10):2828-9.*
59. Horst-Sikorska W, Wawrzyniak A, Celczyńska-Bajew L, Marcinkowska M, Dabrowski S, Kalak R, Słomski R. Polymorphism of VDR gene--the most effective molecular marker of osteoporotic bone fractures risk within postmenopausal women from Wielkopolska region of Poland. *Endokrynol Pol. 2005 May-Jun;56(3):233-9.*
60. Sawicka-Zukowska M, Muszy?ska-Ros?an K, Krawczuk-Rybak M, Galicka A, Panasiuk A [Collagen type I gene polymorphism in children and young adults with neoplastic disease] Polski merkuriusz lekarski : organ Polskiego Towarzystwa Lekarskiego 2008 Mar 24 (141): 237-40.

61. [Association of polymorphisms and haplotypes in the 5' region of COLIA1 gene with the risk of osteoporotic fractures in Russian women from Volga-Ural region] Genetika 2008 Feb 44 (2): 219-25.
Authors are not available
62. Lakatos PL, Bajnok E, Tornai I, Folhoffer A, Horváth A, Lakatos P, Szalay F [Decreased bone mineral density and gene polymorphism in primary biliary cirrhosis]
Orvosi hetilap 2004 Feb 145 (7): 331-6.
63. Brodowska A [The influence of hormonal replacement therapy on bone density in postmenopausal women depending on polymorphism of vitamin D receptor (VDR) and estrogen receptor (ER) genes]
Annales Academiae Medicae Stetinensis 2003 49 (): 111-30.
64. Moskalenko MV, Aseev MV, Zazerskaia IE, Kotova SM, Ivashchenko TE, Baranov VS [Analysis of association of Col1a1 gene alleles with the development of osteoporosis]
Genetika 2002 Dec 38 (12): 1699-703.
65. Krylov MIu, Korotkova TA, Miakotkin VA, Benevolenskaia LI.[Allele polymorphism of alkaline phosphatase, acid soluble phosphatase, and vitamin D-binding protein genes in postmenopausal osteoporosis] Ter Arkh. 2004;76(5):61-5. Russian.
66. Mann V, Ralston SH Meta-analysis of COL1A1 Sp1 polymorphism in relation to bone mineral density and osteoporotic fracture.. *Bone. 2003 Jun;32(6):711-7.*
67. Mann V, Hobson EE, Li B, Stewart TL, Grant SF, Robins SP, Aspden RM, Ralston SH A COL1A1 Sp1 binding site polymorphism predisposes to osteoporotic fracture by affecting bone density and quality.. *J Clin Invest. 2001 Apr;107(7):899-907.*
68. Gong G, Haynatzki G Association between bone mineral density and candidate genes in different ethnic populations and its implications.
Calcified tissue international 2003 Feb 72 (2): 113-23.
69. Ji GR, Yao M, Sun CY, Zhang L, Han Z Association of Collagen Type I alpha1 (COLIA1) Sp1 Polymorphism with Osteoporotic Fracture in Caucasian Post-menopausal Women: a Meta-analysis. The Journal of international medical research 2010 37 (6): 6.
70. Huang QY, Li GH, Cheung WM, Song YQ, Kung AW. Prediction of osteoporosis candidate genes by computational disease-gene identification strategy. J Hum Genet. 2008;53(7):644-55.
71. Uitterlinden AG, Arp PP, Paeper BW, Charmley P, Proll S, Rivadeneira F, Fang Y, van Meurs JB, Britschgi TB, Latham JA, Schatzman RC, Pols HA, Brunkow ME Polymorphisms in the sclerosteosis/van Buchem disease gene (SOST) region are associated with bone-mineral density in elderly whites. American journal of human genetics 2004 Dec 75 (6): 1032-45.

72. Hartikka H, Mäkitie O, Männikkö M, Doria AS, Daneman A, Cole WG, Ala-Kokko L, Sochett EB.Heterozygous mutations in the LDL receptor-related protein 5 (LRP5) gene are associated with primary osteoporosis in children. *J Bone Miner Res. 2005 May;20(5):783-9. Epub 2005 Jan 4.*
73. Giampietro PF, McCarty C, Mukesh B, McKiernan F, Wilson D, Shuldiner A, Liu J, Levasseur J, Ivacic L, Kitchner T, Ghebranious N The role of cigarette smoking and statins in the development of postmenopausal osteoporosis: a pilot study utilizing the Marshfield Clinic Personalized Medicine Cohort. Osteoporosis international : a journal established as result of cooperation between the European Foundation for Osteoporosis and the National Osteoporosis Foundation of the USA 2009 Jun .
74. Mizuguchi T, Furuta I, Watanabe Y, Tsukamoto K, Tomita H, Tsujihata M, Ohta T, Kishino T, Matsumoto N, Minakami H, Niikawa N, Yoshiura K. LRP5, low-density-lipoprotein-receptor-related protein 5, is a determinant for bone mineral density. J Hum Genet. 2004;49(2):80-6.
75. Giedraitis V, Kilander L, Degerman-Gunnarsson M, Sundelöf J, Axelsson T, Syvänen AC, Lannfelt L, Glaser A. Genetic Analysis of Alzheimer's Disease in the Uppsala Longitudinal Study of Adult Men.
Dementia and geriatric cognitive disorders 2009 Jan 27 (1): 1.
76. Simsek M, Cetin Z, Bilgen T, Taskin O, Luleci G, Keser I. Effects of hormone replacement therapy on bone mineral density in Turkish patients with or without COL1A1 Sp1 binding site polymorphism. *J Obstet Gynaecol Res. 2008 Feb;34(1):73-7.*
77. Navarro MC, Sosa M, del Pino-Montes J, Torres A, Salido E, Saavedra P, Corral-Gudino L, Montilla CA.Collagen type 1 (COL1A1) Sp1 binding site polymorphism is associated with osteoporotic fractures but not with bone density in post-menopausal women from the Canary Islands: a preliminary study. *Aging Clin Exp Res. 2007 Feb;19(1):4-9.*
78. Bustamante M, Nogués X, Enjuanes A, Elosua R, García-Giralt N, Pérez-Edo L, Cáceres E, Carreras R, Mellibovsky L, Balcells S, Díez-Pérez A, Grinberg D. COL1A1, ESR1, VDR and TGFB1 polymorphisms and haplotypes in relation to BMD in Spanish postmenopausal women. *Osteoporos Int. 2007 Feb;18(2):235-43. Epub 2006 Oct 5*
79. Suuriniemi M, Kovanen V, Mahonen A, Alén M, Wang Q, Lyytikäinen A, Cheng S.COL1A1 Sp1 polymorphism associates with bone density in early puberty. Bone. 2006 Sep;39(3):591-7. Epub 2006 Mar 31.
80. Yamada Y, Ando F, Niino N, Shimokata H.Association of a -1997G-->T polymorphism of the collagen Ialpha1 gene with bone mineral density in postmenopausal Japanese women. *Hum Biol. 2005 Feb;77(1):27-36.*
81. Todhunter CE, Sutherland-Craggs A, Bartram SA, Donaldson PT, Daly AK, Francis RM, Mansfield JC, Thompson NP.Influence of IL-6, COL1A1, and VDR gene polymorphisms on bone mineral density in Crohn's disease. *Gut. 2005 Nov;54(11):1579-84. Epub 2005 Jul 11.*
82. Bandrés E, Pombo I, González-Huarriz M, Rebollo A, López G, García-Foncillas J. Association between bone mineral density and polymorphisms of the VDR, ERalpha, COL1A1 and CTR genes in Spanish postmenopausal women. *J Endocrinol Invest. 2005 Apr;28(4):312-21.*
83. Liu PY, Lu Y, Long JR, Xu FH, Shen H, Recker RR, Deng HW. Common variants at the PCOL2 and Sp1 binding sites of the COL1A1 gene and their interactive effect influence bone mineral density in Caucasians. J Med Genet. 2004 Oct;41(10):752-7.
84. Ellnebo-Svedlund K, Larsson L, Jonasson J, Magnusson P.Rapid genotyping of the osteoporosis-associated polymorphic transcription factor Sp1 binding site in the COL1A1 gene by pyrosequencing. *Mol Biotechnol. 2004 Jan;26(1):87-90.*

85. McClean E, Archbold GP, Taggart HM Do the COL1A1 and Taq 1 vitamin D receptor polymorphisms have a role in identifying individuals at risk of developing osteoporosis?. *Ulster Med J. 2003 May;72(1):26-33.*
86. Lei SF, Deng FY, Liu XH, Huang QR, Qin Y, Zhou Q, Jiang DK, Li YM, Mo XY, Liu MY, Chen XD, Wu XS, Shen H, Dvornyk V, Zhao L, Recker RR, Deng HW.Polymorphisms of four bone mineral density candidate genes in Chinese populations and comparison with other populations of different ethnicity. *J Bone Miner Metab. 2003;21(1):34-42.*
87. Montanaro L, Arciola CR.Allele frequency of the G-->T mutation of the col1A1 gene analyzed by an ARMS-PCR in osteoporotic subjects with femoral neck fractures. *Clin Chem Lab Med. 2002 Jun;40(6):550-3.*
88. MacDonald HM, McGuigan FA, New SA, Campbell MK, Golden MH, Ralston SH, Reid DM.COL1A1 Sp1 polymorphism predicts perimenopausal and early postmenopausal spinal bone loss. *J Bone Miner Res. 2001 Sep;16(9):1634-41.*
89. Vinkanharju A, Melkko T, Risteli J, Risteli L. New PCR-based method for the Sp1 site polymorphism in the COL1A1 gene. *Clin Chem Lab Med. 2001 Jul;39(7):624-6.*
90. Dennison EM, Arden NK, Keen RW, Syddall H, Day IN, Spector TD, Cooper C. Birthweight, vitamin D receptor genotype and the programming of osteoporosis. *Paediatr Perinat Epidemiol. 2001 Jul;15(3):211-9.*
91. Funke S, Morava E, Czakó M, Vida G, Ertl T, Kosztolányi G Influence of genetic polymorphisms on bone disease of preterm infants.
Pediatric research 2006 Nov 60 (5): 607-12.
92. Stewart TL, Jin H, McGuigan FE, Albagha OM, Garcia-Giralt N, Bassiti A, Grinberg D, Balcells S, Reid DM, Ralston SH Haplotypes defined by promoter and intron 1 polymorphisms of the COLIA1 gene regulate bone mineral density in women. The Journal of clinical endocrinology and metabolism 2006 Sep 91 (9): 3575-83.
93. Ralston SH, Uitterlinden AG, Brandi ML, Balcells S, Langdahl BL, Lips P, Lorenc R, Obermayer-Pietsch B, Scollen S, Bustamante M, Husted LB, Carey AH, Diez-Perez A, Dunning AM, Falchetti A, Karczmarewicz E, Kruk M, van Leeuwen JP, van Meurs JB, Mangion J, McGuigan FE, Mellibovsky L, del Monte F, Pols HA, Reeve J, Reid DM, Renner W, Rivadeneira F, van Schoor NM, Sherlock RE, Ioannidis JP Large-scale evidence for the effect of the COLIA1 Sp1 polymorphism on osteoporosis outcomes: the GENOMOS study.
PLoS medicine 2006 Apr 3 (4): e90.
94. Mezquita-Raya P, Muñoz-Torres M, Alonso G, de Luna JD, Quesada JM, Dorado G, Luque-Recio F, Ruiz-Requena ME, Lopez-Rodriguez F, Escobar-Jiménez F Susceptibility for postmenopausal osteoporosis: interaction between genetic, hormonal and lifestyle factors.
Calcified tissue international 2004 Nov 75 (5): 373-9.
95. Lau EM, Choy DT, Li M, Woo J, Chung T, Sham A The relationship between COLI A1 polymorphisms (Sp 1) and COLI A2 polymorphisms (Eco R1 and Puv II) with bone mineral density in Chinese men and women. Calcified tissue international 2004 Aug 75 (2): 133-7.
96. Pluijm SM, van Essen HW, Bravenboer N, Uitterlinden AG, Smit JH, Pols HA, Lips P Collagen type I alpha1 Sp1 polymorphism, osteoporosis, and intervertebral disc degeneration in older men and women. Annals of the rheumatic diseases 2004 Jan 63 (1): 71-7.
97. Willing MC, Torner JC, Burns TL, Janz KF, Marshall T, Gilmore J, Deschenes SP, Warren JJ, Levy SM Gene polymorphisms, bone mineral density and bone mineral content in young children: the Iowa Bone Development Study. Osteoporosis international : a journal established as result of cooperation between the European Foundation for Osteoporosis and the National Osteoporosis Foundation of the USA 2003 Aug 14 (8): 650-8.

98. Barros ER, Kasamatsu TS, Ramalho AC, Hauache OM, Vieira JG, Lazaretti-Castro M Bone mineral density in young women of the city of São Paulo, Brazil: correlation with both collagen type I alpha 1 gene polymorphism and clinical aspects. Brazilian journal of medical and biological research = Revista brasileira de pesquisas médicas e biológicas / Sociedade Brasileira de Biofísica ... [et al.] 2002 Aug 35 (8): 885-93.
99. Wynne F, Drummond F, O'Sullivan K, Daly M, Shanahan F, Molloy MG, Quane KA Investigation of the genetic influence of the OPG, VDR (Fok1), and COLIA1 Sp1 polymorphisms on BMD in the Irish population.
Calcified tissue international 2002 Jul 71 (1): 26-35.
100. Arisal O, Deviren A, Fenerci EY, Hacihanefioglu S, Ulutin T, Erkmen S, Buyru N Polymorphism analysis in the COLIA1 gene of patients with thalassemia major and intermedia. Haematologia 2002 32 (4): 475-82.
101. Braga V, Sangalli A, Malerba G, Mottes M, Mirandola S, Gatti D, Rossini M, Zamboni M, Adami S Relationship among VDR (BsmI and FokI), COLIA1, and CTR polymorphisms with bone mass, bone turnover markers, and sex hormones in men. Calcified tissue international 2002 Jun 70 (6): 457-62.
102. The collagen Ia1 SP1 polymorphism is associated with differences in ultrasound transmission velocity in the calcaneus in postmenopausal women. Calcified tissue international 2002 Jun 70 (6): 450-6.
Kann P, Bergink AP, Fang Y, Van Daele PL, Hofman A, Van Leeuwen JP, Beyer J, Uitterlinden AG, Pols HA
103. Qureshi AM, Herd RJ, Blake GM, Fogelman I, Ralston SH COLIA1 Sp1 polymorphism predicts response of femoral neck bone density to cyclical etidronate therapy. Calcified tissue international 2002 Mar 70 (3): 158-63.
104. Van Pottelbergh I, Goemaere S, Nuytinck L, De Paepe A, Kaufman JM Association of the type I collagen alpha1 Sp1 polymorphism, bone density and upper limb muscle strength in community-dwelling elderly men. Osteoporosis international : a journal established as result of cooperation between the European Foundation for Osteoporosis and the National Osteoporosis Foundation of the USA 2001 12 (10): 895-901.
105. Lambrinoudaki I, Kung AW Absence of high-risk "s" allele associated with osteoporosis at the intronic SP1 binding-site of collagen Ialpha1 gene in Southern Chinese. Journal of endocrinological investigation 2001 24 (7): 499-502.

106. Ashford RU, Luchetti M, McCloskey EV, Gray RL, Pande KC, Dey A, Kayan K, Ralston SH, Kanis JA Studies of bone density, quantitative ultrasound, and vertebral fractures in relation to collagen type I alpha 1 alleles in elderly women. Calcified tissue international 2001 Jun 68 (6): 348-51.

107. Efstathiadou Z, Kranas V, Ioannidis JP, Georgiou I, Tsatsoulis A The Sp1 COLIA1 gene polymorphism, and not vitamin D receptor or estrogen receptor gene polymorphisms, determines bone mineral density in postmenopausal Greek women. Osteoporosis international : a journal established as result of cooperation between the European Foundation for Osteoporosis and the National Osteoporosis Foundation of the USA 2001 12 (4): 326-31.
108. Sheehan D, Bennett T, Cashman KD An assessment of genetic markers as predictors of bone turnover in healthy adults. Journal of endocrinological investigation 2001 Apr 24 (4): 236-45.

109. Parés A, Guañabens N, Alvarez L, De Osaba MJ, Oriola J, Pons F, Caballería L, Monegal A, Salvador G, Jo J, Peris P, Rivera F, Ballesta AM, Rodés J Collagen type Ialpha1 and vitamin D receptor gene olymorphisms and bone mass in primary biliary cirrhosis. Hepatology (Baltimore, Md.) 2001 Mar 33 (3):

554-60.

110. Braga V, Mottes M, Mirandola S, Lisi V, Malerba G, Sartori L, Bianchi G, Gatti D, Rossini M, Bianchini D, Adami S Association of CTR and COLIA1 alleles with BMD values in peri- and postmenopausal women. Calcified tissue international 2000 Nov 67 (5): 361-366.

111. Mencej-Bedrac S, Prezelj J, Kocjan T, Komadina R, Marc J.Analysis of association of LRP5, LRP6, SOST, DKK1, and CTNNB1 genes with bone mineral density in a Slovenian population. Calcif Tissue Int. 2009 Dec;85(6):501-6.

112. Dennison EM, Syddall HE, Jameson KA, Sayer AA, Gaunt TR, Rodriguez S, Day IN, Cooper C, Lips MA; Hertfordshire Cohort Study Group.A study of relationships between single nucleotide polymorphisms from the growth hormone-insulin-like growth factor axis and bone mass: the Hertfordshire cohort study. J Rheumatol. 2009 Jul;36(7):1520-6.

113. Lazáry A, Kósa JP, Tóbiás B, Lazáry J, Balla B, Bácsi K, Takács I, Nagy Z, Mezo T, Speer G, Lakatos P.Single nucleotide polymorphisms in new candidate genes are associated with bone mineral density and fracture risk. Eur J Endocrinol. 2008 Aug;159(2):187-96.

114. Lee HJ, Kim SY, Koh JM, Bok J, Kim KJ, Kim KS, Park MH, Shin HD, Park BL, Kim TH, Hong JM, Park EK, Kim DJ, Oh B, Kimm K, Kim GS, Lee JY.Polymorphisms and haplotypes of integrinalpha1 (ITGA1) are associated with bone mineral density and fracture risk in postmenopausal Koreans. Bone. 2007 Dec;41(6):979-86.

115. Rivadeneira F, van Meurs JB, Kant J, Zillikens MC, Stolk L, Beck TJ, Arp P, Schuit SC, Hofman A, Houwing-Duistermaat JJ, van Duijn CM, van Leeuwen JP, Pols HA, Uitterlinden AG Estrogen receptor beta (ESR2) polymorphisms in interaction with estrogen receptor alpha (ESR1) and insulin-like growth factor I (IGF1) variants influence the risk of fracture in postmenopausal women.. J Bone Miner Res. 2006 Sep;21(9):1443-56.

116. Lei SF, Zhang YY, Deng FY, Liu MY, Liu XH, Zhou XG, Deng HW. Bone mineral density and five prominent candidate genes in Chinese men: associations, interaction effects and their implications. Maturitas. 2005 Jun 16;51(2):199-206.

117. Lau EM, Choy DT, Li M, Woo J, Chung T, Sham A. The relationship between COLI A1 polymorphisms (Sp 1) and COLI A2 polymorphisms (Eco R1 and Puv II) with bone mineral density in Chinese men and women. Calcif Tissue Int. 2004 Aug;75(2):133-7.

118. Suuriniemi M, Mahonen A, Kovanen V, Alén M, Cheng S. Relation of PvuII site polymorphism in the COL1A2 gene to the risk of fractures in prepubertal Finnish girls. Physiol Genomics. 2003 Aug 15;14(3):217-24.

119. Watanabe Y, Kinoshita A, Yamada T, Ohta T, Kishino T, Matsumoto N, Ishikawa M, Niikawa N, Yoshiura K. A catalog of 106 single-nucleotide polymorphisms (SNPs) and 11 other types of variations in genes for transforming growth factor-beta1 (TGF-beta1) and its signaling pathway. J Hum Genet. 2002;47(9):478-83.

120. Walker LM, Preston MR, Magnay JL, Thomas PB, El Haj AJ.Nicotinic regulation of c-fos and osteopontin expression in human-derived osteoblast-like cells and human trabecular bone organ culture. Bone. 2001 Jun;28(6):603-8.

121. McGuigan FE, Murray L, Gallagher A, Davey-Smith G, Neville CE, Van't Hof R, Boreham C, Ralston SH Genetic and environmental determinants of peak bone mass in young men and women.
Journal of bone and mineral research : the official journal of the American Society for Bone and Mineral Research 2002 Jul 17 (7): 1273-9.

122. Breuil V, Quincey D, Testa J, Roux CH, Albert C, Mroueh Z, Chami-Stemman H, Brocq O, Grisot C, Euller-Ziegler L, Carle GF Gene polymorphisms and osteoporotic fractures: A study in postmenopausal French women. Joint, bone, spine : revue du rhumatisme 2009 Mar .

123. Gerdhem P, Brändström H, Stiger F, Obrant K, Melhus H, Ljunggren O, Kindmark A, Akesson K Association of the collagen type 1 (COL1A 1) Sp1 binding site polymorphism to femoral neck bone mineral density and wrist fracture in 1044 elderly Swedish women.
Calcified tissue international 2004 Mar 74 (3): 264-9.

124. Hubacek JA, Weichetova M, Bohuslavova R, Skodova Z, Stepan JJ, Adamkova V. No associations between genetic polymorphisms of TGF-beta, PAI-1, and COL1A1, and bone mineral density in Caucasian females. Endocr Regul. 2006 Dec;40(4):107-12.

125. Hubacek JA, Weichetova M, Bohuslavova R, Skodova Z, Adámkova V, Stepan JJ Genetic polymorphisms of TGF-beta, PAI-1, and COL1A-1, and determination of bone mineral density in Caucasian females. Endocrine regulations 2006 Sep 40 (3): 77-81.

126. Castellani C, Malerba G, Sangalli A, Delmarco A, Petrelli E, Rossini M, Assael BM, Mottes M. The genetic background of osteoporosis in cystic fibrosis: association analysis with polymorphic markers in four candidate genes. *J Cyst Fibros. 2006 Dec; 5(4):229-35. Epub 2006 May 18.*

127. Xu FH, Liu YJ, Deng H, Huang QY, Zhao LJ, Shen H, Liu YZ, Dvornyk V, Conway T, Li JL, Davies KM, Recker RR, Deng HW.A follow-up linkage study for bone size variation in an extended sample. *Bone. 2004 Sep;35(3):777-84.*

128. Long JR, Zhao LJ, Liu PY, Lu Y, Dvornyk V, Shen H, Liu YJ, Zhang YY, Xiong DH, Xiao P, Deng HW. Patterns of linkage disequilibrium and haplotype distribution in disease candidate genes. *BMC Genet. 2004 May 24;5:11.*

129. McClean E, Archbold GP, Taggart HM. Do the COL1A1 and Taq 1 vitamin D receptor polymorphisms have a role in identifying individuals at risk of developing osteoporosis? *Ulster Med J. 2003 May;72(1):26-33.*

130. Collaborative Richards JB, Kavvoura FK, Rivadeneira F, Styrkársdóttir U, Estrada K, Halldórsson BV, Hsu YH, Zillikens MC, Wilson SG, Mullin BH, Amin N, Aulchenko YS, Cupples LA, Deloukas P, Demissie S, Hofman A, Kong A, Karasik D, van Meurs JB, Oostra BA, Pols HA, Sigurdsson G, Thorsteinsdottir U, Soranzo N, Williams FM, Zhou Y, Ralston SH, Thorleifsson G, van Duijn CM, Kiel DP, Stefansson K, Uitterlinden AG, Ioannidis JP, Spector TD; Genetic Factors for Osteoporosis Consortium. meta-analysis: associations of 150 candidate genes with osteoporosis and osteoporotic fracture. Ann Intern Med. 2009 Oct 20;151(8):528-37.

131. Rivadeneira F, Styrkársdottir U, Estrada K, Halldórsson BV, Hsu YH, Richards JB, Zillikens MC, Kavvoura FK, Amin N, Aulchenko YS, Cupples LA, Deloukas P, Demissie S, Grundberg E, Hofman A, Kong A, Karasik D, van Meurs JB, Oostra B, Pastinen T, Pols HA, Sigurdsson G, Soranzo N, Thorleifsson G, Thorsteinsdottir U, Williams FM, Wilson SG, Zhou Y, Ralston SH, van Duijn CM, Spector T, Kiel DP, Stefansson K, Ioannidis JP, Uitterlinden AG; Genetic Factors for Osteoporosis (GEFOS) Consortium.Twenty bone-mineral-density loci identified by large-scale meta-analysis of genome-wide association studies. Nat Genet. 2009 Nov;41(11):1199-206.

132. Tran BN, Nguyen ND, Center JR, Eisman JA, Nguyen TV Enhancement of Absolute Fracture Risk Prognosis with Genetic Marker: The Collagen I Alpha 1 Gene.
Calcified tissue international 2009 Sep .

133. Jin H, van't Hof RJ, Albagha OM, Ralston SH. Promoter and intron 1 polymorphisms of COL1A1 interact to regulate transcription and susceptibility to osteoporosis. *Hum Mol Genet. 2009 Aug 1;18(15):2729-38. Epub 2009 May 9.*
134. Musumeci M, Vadalà G, Tringali G, Insirello E, Roccazzello AM, Simpore J, Musumeci S. Genetic and environmental factors in human osteoporosis from Sub-Saharan to Mediterranean areas. *J Bone Miner Metab. 2009;27(4):424-34. Epub 2009 Mar 3.*
135. Dinçel E, Sepici-Dinçel A, Sepici V, Ozsoy H, Sepici B. Hip fracture risk and different gene polymorphisms in the Turkish population. *Clinics (Sao Paulo). 2008 Oct;63(5):645-50.*
136. Peris P, Alvarez L, Oriola J, Guañabens N, Monegal A, de Osaba MJ, Jo J, Pons F, Ballesta AM, Muñoz-Gómez J. Collagen type Ialpha1 gene polymorphism in idiopathic osteoporosis in men. *Rheumatology (Oxford). 2000 Nov;39(11):1222-5.*
137. McGuigan FE, Reid DM, Ralston SH. Susceptibility to osteoporotic fracture is determined by allelic variation at the Sp1 site, rather than other polymorphic sites at the COL1A1 locus. *Osteoporos Int. 2000;11(4):338-43.*
138. Keen RW, Woodford-Richens KL, Grant SF, Ralston SH, Lanchbury JS, Spector TD. Association of polymorphism at the type I collagen (COL1A1) locus with reduced bone mineral density, increased fracture risk, and increased collagen turnover. *Arthritis Rheum. 1999 Feb;42(2):285-90.*
139. Willing MC, Torner JC, Burns TL, Segar ET, Werner JR.Determinants of bone mineral density in postmenopausal white Iowans. *J Gerontol A Biol Sci Med Sci. 1997 Nov;52(6):M337-42..*
140. Falcón-Ramírez E, Casas-Avila L, Miranda A, Diez P, Castro C, Rubio J, Gómez R, Valdés-Flores M Sp1 polymorphism in collagen I alpha1 gene is associated with osteoporosis in lumbar spine of Mexican women. Molecular biology reports 2010 Feb .
141. Husted LB, Harsløf T, Gonzalez-Bofill N, Schmitz A, Carstens M, Stenkjær L, Langdahl BL Haplotypes of Promoter and Intron 1 Polymorphisms in the COLIA1 Gene Are Associated with Increased Risk of Osteoporosis. Calcified tissue international 2008 Dec .
142. Guzeloglu-Kayisli O, Cetin Z, Keser I, Ozturk Z, Tuncer T, Canatan D, Luleci G Relationship between SP1 polymorphism and osteoporosis in beta-thalassemia major patients. Pediatrics international : official journal of the Japan Pediatric Society 2008 Aug 50 (4): 4.
143. Weichetova M, Stepan JJ, Haas T, Michalska D The risk of Colles' fracture is associated with the collagen I alpha1 Sp1 polymorphism and ultrasound transmission velocity in the calcaneus only in heavier postmenopausal women. Calcified tissue international 2005 Feb 76 (2): 98-106.
144. Lakatos PL, Bajnok E, Tornai I, Folhoffer A, Horvath A, Lakatos P, Habior A, Szalay F Insulin-like growth factor I gene microsatellite repeat, collagen type Ialpha1 gene Sp1 polymorphism, and bone disease in primary biliary cirrhosis. European journal of gastroenterology & hepatology 2004 Aug 16 (8): 753-9.
145. Alvarez-Hernández D, Naves M, Díaz-López JB, Gómez C, Santamaría I, Cannata-Andía JB Influence of polymorphisms in VDR and COLIA1 genes on the risk of osteoporotic fractures in aged men.
Kidney international. Supplement 2003 Jun (85): S14-8.
146. Mezquita-Raya P, Muñoz-Torres M, de Dios Luna J, Lopez-Rodriguez F, Quesada JM, Luque-Recio F, Escobar-Jiménez F Performance of COLIA1 polymorphism and bone turnover markers to identify postmenopausal women with prevalent vertebral fractures.
Osteoporosis international : a journal established as result of cooperation between

the European Foundation for Osteoporosis and the National Osteoporosis Foundation of the USA 2002 13 (6): 506-12.
147. Bernad M, Martinez ME, Escalona M, González ML, González C, Garcés MV, Del Campo MT, Martín Mola E, Maderò R, Carreñò L Polymorphism in the type I collagen (COLIA1) gene and risk of fractures in postmenopausal women. Bone 2002 Jan 30 (1): 223-8.
148. Qureshi AM, McGuigan FE, Seymour DG, Hutchison JD, Reid DM, Ralston SH Association between COLIA1 Sp1 alleles and femoral neck geometry. Calcified tissue international 2001 Aug 69 (2): 67-72.
149. McGuigan FE, Armbrecht G, Smith R, Felsenberg D, Reid DM, Ralston SH Prediction of osteoporotic fractures by bone densitometry and COLIA1 genotyping: a prospective, population-based study in men and women. Osteoporosis international : a journal established as result of cooperation between the European Foundation for Osteoporosis and the National Osteoporosis Foundation of the USA 2001 12 (2): 91-6.
150. Uitterlinden AG, Weel AE, Burger H, Fang Y, van Duijn CM, Hofman A, van Leeuwen JP, Pols HA Interaction between the vitamin D receptor gene and collagen type Ialpha1 gene in susceptibility for fracture.
Journal of bone and mineral research : the official journal of the American Society for Bone and Mineral Research 2001 Feb 16 (2): 379-85.
151. Perrotta S, Cappellini MD, Bertoldo F, Servedio V, Iolascon G, D'Agruma L, Gasparini P, Siciliani MC, Iolascon A Osteoporosis in beta-thalassaemia major patients: analysis of the genetic background. British journal of haematology 2000 Nov 111 (2): 461-6.
152. Aerssens J, Dequeker J, Peeters J, Breemans S, Broos P, Boonen S.Polymorphisms of the VDR, ER and COLIA1 genes and osteoporotic hip fracture in elderly postmenopausal women. Osteoporos Int. 2000;11(7):583-91.
153. Lidén M, Wilén B, Ljunghall S, Melhus H. Polymorphism at the Sp 1 binding site in the collagen type I alpha 1 gene does not predict bone mineral density in postmenopausal women in sweden. Calcif Tissue Int. 1998 Oct;63(4):293-5.
154. Roux C, Dougados M, Abel L, Mercier G, Lucotte G. Association of a polymorphism in the collagen I alpha1 gene with osteoporosis in French women. Arthritis Rheum. 1998 Jan;41(1):187-8.
155. Nakajima T, Ota N, Shirai Y, Hata A, Yoshida H, Suzuki T, Hosoi T, Orimo H, Emi M.Ethnic difference in contribution of Sp1 site variation of COLIA1 gene in genetic predisposition to osteoporosis. Calcif Tissue Int. 1999 Nov;65(5):352-3.
156. Alvarez L, Oriola J, Jo J, Ferró T, Pons F, Peris P, Guañabens N, Durán M, Monegal A, Martínez de Osaba MJ, Rivera-Fillat F, Ballesta AM. Collagen type I alpha1 gene Sp1 polymorphism in premenopausal women with primary osteoporosis: improved detection of Sp1 binding site polymorphism in the collagen type 1 gene. Clin Chem. 1999 Jun;45(6 Pt 1):904-6.
157. Langdahl BL, Ralston SH, Grant SF, Eriksen EF. An Sp1 binding site polymorphism in the COLIA1 gene predicts osteoporotic fractures in both men and women. J Bone Miner Res. 1998 Sep;13(9):1384-9.
158. Grant SF, Reid DM, Blake G, Herd R, Fogelman I, Ralston SH. Reduced bone density and osteoporosis associated with a polymorphic Sp1 binding site in the collagen type I alpha 1 gene. Nat Genet. 1996 Oct;14(2):203-5.
159. . Välimäki S, Tähtelä R, Kainulainen K, Laitinen K, Löyttyniemi E, Sulkava R, Välimäki M, Kontula KRelation of collagen type I alpha 1 (COLIA 1) and vitamin D receptor genotypes to bone mass, turnover, and fractures in early postmenopausal women and to hip fractures in elderly people. Eur J Intern Med. 2001 Feb;12(1):48-56.

160. Rosen CJ, Kurland ES, Vereault D, Adler RA, Rackoff PJ, Craig WY, Witte S, Rogers J, Bilezikian JP.Association between serum insulin growth factor-I (IGF-I) and a simple sequence repeat in IGF-I gene: implications for genetic studies of bone mineral density. J Clin Endocrinol Metab. 1998 Jul;83(7):2286-90.
161. Kim JG, Roh KR, Lee JY.The relationship among serum insulin-like growth factor-I, insulin-like growth factor-I gene polymorphism, and bone mineral density in postmenopausal women in Korea. Am J Obstet Gynecol. 2002 Mar;186(3):345-50.
162. Delmonico MJ, Zmuda JM, Taylor BC, Cauley JA, Harris TB, Manini TM, Schwartz A, Li R, Roth SM, Hurley BF, Bauer DC, Ferrell RE, Newman AB; Health ABC and MrOS Research Groups.Association of the ACTN3 genotype and physical functioning with age in older adults. J Gerontol A Biol Sci Med Sci. 2008 Nov;63(11):1227-34.
163. Haussler MR, Haussler CA, Whitfield GK, Hsieh JC, Thompson PD, Barthel TK, Bartik L, Egan JB, Wu Y, Kubicek JL, Lowmiller CL, Moffet EW, Forster RE, Jurutka PW. The nuclear vitamin D receptor controls the expression of genes encoding factors which feed the "Fountain of Youth" to mediate healthful aging. J Steroid Biochem Mol Biol. 2010 Mar 20.
164. Xiao Y, Cui J, Li YX, Shi YH, Le GW. Expression of genes associated with bone resorption is increased and bone formation is decreased in mice fed a high-fat diet. Lipids. 2010 Apr;45(4):345-55.
165. Erdogan MO, Yıldız H, Artan S, Solak M, Taşçıoğlu F, Dündar U, Eser B, Colak E. Association of estrogen receptor alpha and collagen type I alpha 1 gene polymorphisms with bone mineral density in postmenopausal women. Osteoporos Int. 2010 Jun 8.
166. Tofteng CL, Bach-Mortensen P, Bojesen SE, Tybjaerg-Hansen A, Hyldstrup L, Nordestgaard BG. Integrin beta3 Leu33Pro polymorphism and risk of hip fracture: 25 years follow-up of 9233 adults from the general population.Pharmacogenet Genomics. 2007 Jan;17(1):85-91.
167. Liu YZ, Pei YF, Liu JF, Yang F, Guo Y, Zhang L, Liu XG, Yan H, Wang L, Zhang YP, Levy S, Recker RR, Deng HW. 2009 Powerful bivariate genome-wide association analyses suggest the SOX6 gene influencing both obesity and osteoporosis phenotypes in males. PLoS One. Aug 28;4(8):e6827.
168. Kiel DP, Demissie S, Dupuis J, Lunetta KL, Murabito JM, Karasik D. 2007Genome-wide association with bone mass and geometry in the Framingham Heart Study. BMC Med Genet. Sep 19;8 Suppl 1:S14.
169. Lee DO, Jee BC, Ku SY, Suh CS, Kim SH, Choi YM, Moon SY, Kim JG. Relationships between the insulin-like growth factor I (IGF-I) receptor gene G3174A polymorphism, serum IGF-I levels, and bone mineral density in postmenopausal Korean women. J Bone Miner Metab. 2008;26(1):42-6.
