# Supplementary material for: Synopsis and meta-analysis of genetic association studies in osteoporosis for the focal adhesion family genes: the CUMAGAS-OSTEOporosis information system
Source: BMC Med. 2011 Jan 26;9:9. doi: 10.1186/1741-7015-9-9 (PMC3040157; doi:10.1186/1741-7015-9-9)
Supplement: Additional file 2 — Supplementary Table 2 [file 1741-7015-9-9-S2.PDF]

					Cases							Controls							Allele contrast			Recessive model			Dominant model			Additive model			Co-dominant model		
**Gene**	**Variant**	**Author**	**Year**	**ethnicity**	**N**	**Age yrs (Mean±SD) (min-max)**	**gender**	**menopause status**	**BMD site**	**Diagnosis**	**Matching**	**N**	**Health status**	**Age (Mean±SD)yrs (min-max)yrs**	**Menopause status**	**BMD site**	**HWE**	**Power**	**OR**	**95% LL**	**95% UL**	**OR**	**95% LL**	**95% UL**	**OR**	**95% LL**	**95% UL**	**OR**	**95% LL**	**95% UL**	**OR**	**95% LL**	**95% UL**
COL1A1	Sp1 SS/ss G2046T (rs1800012)	Grant	1996	Whites	55	61.3±0.74*	female	87.3% postmenopausal	spine, femoral neck	vertebral compression fracture	age, sex	55	healthy	55±0.7	postmenopausal	spine, femoral neck	yes	0.06	2.60	1.31	5.14	7.40	0.37	146.74	2.97	1.34	6.59	10.70	0.53	215.62	2.39	1.08	5.30
	Sp1 SS/ss G2046T (rs1800012)	Liden	1998	Whites	64	68.7±8.1	female	postmenopausal	lumbar spine (L2-L4), femoral neck	osteoporotic	age, weight, height, BMI	72	healthy	66. 8±9.7	postmenopausal	lumbar spine(L2-L4), femoral neck	yes	0.06	0.84	0.44	1.59	0.56	0.05	6.28	0.84	0.41	1.75	0.53	0.05	6.09	0.89	0.42	1.86
	Sp1 SS/ss G2046T (rs1800012)	Nakajima	1998	Japanese	202	72.4±5.6	female	postmenopausal	radius	osteoporosis (criteria of Japanese society for bone and mineral research)		202	normal	68.7±6.36	NR	radius	not	0.03	1.00	0.02	50.52	1.00	0.02	50.64	1.00	0.02	50.64	1.00	0.02	50.64	1.00	0.02	50.64
	Sp1 SS/ss G2046T (rs1800012)	Roux	1998	Whites	110	63.1±12.3 (45-90)	female	postmenopausal	lumbar spine, femoral neck	osteoporotic and osteopenic		107	healthy	NR	NA (male & female)	lumbar spine, femoral neck	yes	0.08	1.66	0.99	2.78	0.97	0.13	7.03	1.92	1.07	3.46	1.19	0.16	8.68	1.98	1.09	3.59
	Sp1 SS/ss G2046T (rs1800012)	Langdahl (female)	1998	Whites	77	58.3± 6.9	female	NR	lumbar spine, femoral neck	severe osteoporosis (≥1 nontraumatic fracture of the spine)	age, sex	77	healthy	56.1±7.8	NR	lumbar spine, femoral neck	yes	0.08	1.61	0.96	2.70	10.06	1.24	81.47	1.37	0.73	2.59	10.38	1.26	85.66	0.90	0.47	1.71
	Sp1 SS/ss G2046T (rs1800012)	Langdahl (male)	1998	Whites	28	55.4±11.1	male	NA (male)	lumbar spine, femoral neck	severe osteoporosis (≥1 nontraumatic fracture of the spine)	age, sex	67	healthy	52.2±15.8	NA (male)	lumbar spine, femoral neck	yes	0.06	2.87	1.37	6.02	18.00	2.05	157.86	2.04	0.81	5.14	18.38	2.05	164.34	0.80	0.28	2.31
	Sp1 SS/ss G2046T (rs1800012)	Alvarez	1999	Whites	20	40±9 (25-52)	female	premenopausal	lumbar spine	premenopausal primary osteoporosis	NR	24	healthy	43±7 (27-52)	premenopausal	lumbar spine	yes	0.05	5.69	1.46	22.15	3.77	0.15	97.74	7.00	1.57	31.18	6.14	0.23	164.01	5.73	1.28	25.58
	Sp1 SS/ss G2046T (rs1800012)	Keen	1999	Whites	55	56.4±5.2	female	pre/post- menopausal	lumbar spine, femoral neck	fracture	age, BMI	130	no fractures	53.4±4.0	pre/post- menopausal	lumbar spine, femoral neck	NA								1.95	1.03	3.71						
	Sp1 SS/ss G2046T (rs1800012)	Peris	2000	Whites	35	50.4±10.3 (31-71)	male	NA (male)	lumbar spine (L2-L4) or atraumatic fractures	idiopathic male osteoporosis		60	healthy males	47±17	men	lumbar spine (L2-L4)	yes	0.06	3.29	1.52	7.14	3.58	0.31	40.94	4.24	1.69	10.59	5.65	0.48	66.32	3.75	1.48	9.53
	Sp1 SS/ss G2046T (rs1800012)	Aerssens	2000	Whites	135	78±9	female	postmenopausal	femoral neck, lumbar spine	hip fracture	ethnicity	239	free from diseases affecting bone metabolism	76±4 (70-90)	postmenopausal	femoral neck, lumbar spine	yes	0.11	0.81	0.55	1.18	0.82	0.32	2.06	0.77	0.49	1.21	0.76	0.30	1.93	0.80	0.50	1.28
	Sp1 SS/ss G2046T (rs1800012)	Weichetova	2000	Whites	126	62.2±6.0	female	postmenopausal	femoral neck, lumbar spine	wrist fracture		126	osteoporosis or osteopenia without fracture	60.8±5.8	postmenopausal	femoral neck, lumbar spine	yes	0.09	1.75	1.09	2.80	3.65	0.74	17.91	1.75	1.02	3.00	4.16	0.84	20.62	1.49	0.85	2.59
	Sp1 SS/ss G2046T (rs1800012)	McGuigan	2000	Whites	93	70.2±5.6	both	NA (male & female)	lumbar spine	vertabral fracture	age, sex	88	healthy	71.4±6.2	NA (male & female)	lumbar spine	yes	0.08	2.64	1.47	4.72	6.00	0.71	50.88	2.81	1.45	5.44	7.78	0.91	66.54	2.30	1.17	4.53
	Sp1 SS/ss G2046T (rs1800012)	Perrota	2000	Whites	135	24.0±0.4	both	NA (male & female)	lumbar spine	β-thalassemia and osteoporosis		24	β-thalassemia	NR	NR	lumbar spine	yes	0.04	4.77	1.10	20.68	1.63	0.09	30.50	5.71	1.23	26.60	2.60	0.14	49.18	4.68	1.00	21.80
	Sp1 SS/ss G2046T (rs1800012)	Qureshi	2001	Whites	153	82.75±1.3	female	NR	femoral neck, hip axis	osteoporotic hip fracture		183	healthy	66.5±2.86 (55-87)	NR	left hip	yes	0.10	1.32	0.89	1.96	1.51	0.40	5.73	1.38	0.88	2.18	1.67	0.44	6.39	1.34	0.84	2.12
	Sp1 SS/ss G2046T (rs1800012)	McGuigan (female)	2001	Whites	30	62.8±0.56	female	postmenopausal	lumbar spine, hip	fracture	weight, height	155	no fractures	62.4±0.61	postmenopausal	lumbar spine, hip	yes	0.07	2.45	1.28	4.69	17.11	1.72	170.65	2.35	1.06	5.24	21.19	2.08	216.24	1.61	0.71	3.67
	Sp1 SS/ss G2046T (rs1800012)	McGuigan (male)	2001	Whites	9	59.8±2.1	male	NA (male)	lumbar spine, hip	fracture	weight, height	147	no fractures	64.8±0.71	NA (male)	lumbar spine, hip	yes	0.05	1.01	0.32	3.16	1.71	0.19	15.09	0.84	0.20	3.48	1.53	0.17	14.05	0.65	0.13	3.24
	Sp1 SS/ss G2046T (rs1800012)	Valimaki (early post.)	2001	Whites	402	53±3	female	early postmenopausal	lumbar spine, femoral neck	osteopenia	age	111	healthy	53±3	postmenopausal	lumbar spine, femoral neck	yes	0.09	1.23	0.81	1.85	1.01	0.28	3.70	1.31	0.82	2.08	1.10	0.30	4.02	1.32	0.82	2.15
	Sp1 SS/ss G2046T (rs1800012)	Valimaki (elderly)	2001	Whites	64	89 (95-98)	both	NA (male & female)		hip fracture	age, sex	108	no hip fracture	89 (85-98)	NA (male & female)		yes	0.06	1.50	0.75	2.98	16.14	0.85	304.91	1.12	0.51	2.44	15.47	0.82	293.11	0.72	0.31	1.69
	Sp1 SS/ss G2046T (rs1800012)	Uitterlinden	2001	Whites	97	55-80	female	postmenopausal	lumbar spine, femoral neck	fracture		907	no fractures	55-80	postmenopausal	lumbar spine, femoral neck	yes	0.13	1.76	1.25	2.48	2.74	1.15	6.52	1.85	1.21	2.83	3.31	1.37	8.00	1.57	1.02	2.42
	Sp1 SS/ss G2046T (rs1800012)	Bernad	2002	Whites	221	60.44±5.71	female	postmenopausal	femoral neck, lumbar spine	osteoporosis		98	non oosteoporotic	57.4± 6.2	postmenopausal	lumbar spine, femoral neck	yes	0.10	1.18	0.82	1.71	2.41	0.97	5.99	0.99	0.61	1.59	2.20	0.86	5.64	0.72	0.45	1.17
	Sp1 SS/ss G2046T (rs1800012)	Mezquita-Raya	2002	Whites	43	63±7	female	postmenopausal	femoral neck, lumbar spine	vertabral fracture		101	osteoporotic without fracture	60±7	postmenopausal	lumbar spine, femoral neck	yes	0.07	1.94	1.13	3.31	2.49	0.59	10.44	2.65	1.24	5.67	4.15	0.92	18.85	2.06	1.00	4.27
	Sp1 SS/ss G2046T (rs1800012)	Alvarez-Hernandez	2003	Whites	17	64±9 (50 -86)	male	NA (male)	femoral neck, lumbar spine	vertabral fracture		116	non- fractured	64±9 (50 -86)	NA (male)	lumbar spine, femoral neck	yes	0.06	2.15	0.99	4.65	7.64	2.02	28.83	1.28	0.45	3.62	6.25	1.61	24.30	0.31	0.07	1.42
	Sp1 SS/ss G2046T (rs1800012)	Lakatos	2004	Whites	22	60.8±8.8	female	pre/post- menopausal	lumbar spine, femoral neck	primary biliary cirrhosis & osteoporosis		48	pimary biliary cirrhosis without osteoporosis	56.2±11.1	pre/post- menopausal	lumbar spine, femoral neck	yes	0.05	2.06	0.81	5.21	4.70	0.40	54.84	1.92	0.64	5.77	5.29	0.44	62.99	1.43	0.44	4.58
	Sp1 SS/ss G2046T (rs1800012)	Gerdhem	2004	Whites	420	75.2± 0.1 (75.01-75.99)	female	postmenopausal	femoral neck, lumbar spine	any fracture		544	no fractures	75.2±0.1 (75.01-75.99)	postmenopausal	femoral neck, lumbar spine	yes	0.20	1.22	0.95	1.55	1.79	0.82	3.95	1.20	0.91	1.58	1.87	0.84	4.12	1.12	0.84	1.49
	Sp1 SS/ss G2046T (rs1800012)	Hubacek	2006	Whites	218	58.7 (40-70)	female	postmenopausal	lumbar spine, hip	osteoporotic (23.4% fractures)		151	healthy	59.1 (40-70)	postmenopausal		NA								1.09	0.71	1.68						
	Sp1 SS/ss G2046T (rs1800012)	Musumeci	2009	Whites	100	49.9±3.1 (55-70)	female	postmenopausal	proximal femur	osteoporosis	ethnicity	100	healthy	52.39±4.38 (45-65)	postmenopausal	proximal femur	yes	0.09	1.66	1.09	2.55	2.76	1.03	7.45	1.69	0.97	2.96	3.31	1.18	9.30	1.18	0.67	2.06
	Sp1 SS/ss G2046T (rs1800012)	Meadeb	2008	Whites	92	70±7.4	female	postmenopausal	spine and femoral neck	primary osteoporosis		69	without osteoporosis, fractures or preventive treatment	64.1±7.7	postmenopausal	spine and femoral neck	yes	0.08	1.07	0.66	1.74	1.13	0.31	4.18	1.09	0.58	2.04	1.18	0.31	4.52	1.06	0.57	1.98
	Sp1 SS/ss G2046T (rs1800012)	Guzeloglu- Kayisli	2008	Turkish	37	13.5±3.5	both	NA (children)	lumbar spine, femur	β-thalassemia and osteoporosis		92	healthy	25.4±5.0	NR	lumbar spine, femur	yes	0.07	10.11	5.03	20.32	101.94	5.85	1775.81	7.53	3.19	17.76	135.00	7.62	2392.75	1.65	0.65	4.19
	Sp1 SS/ss G2046T (rs1800012)	Dincel	2008	Turkish	21	74.47±8.91	both	NR	intact side of hip	femur fracture due to simple low-energy tauma		21	osteoporotic without fracture	75.47±7.44	NR	right side of hip	yes	0.05	1.68	0.67	4.22	1.69	0.25	11.42	2.29	0.59	8.82	2.70	0.33	21.98	1.68	0.47	5.97
	Sp1 SS/ss G2046T (rs1800012)	Husted (female)	2009	Whites	228	65.1±8.2	female	pre/post- menopausal	lumbar spine, femoral neck	osteoporosis	age, BMI	226	healthy	65.3±8.2	pre/post- menopausal	lumbar spine, femoral neck	yes	0.12	1.11	0.78	1.57	1.49	0.52	4.27	1.08	0.73	1.61	1.51	0.52	4.34	1.02	0.68	1.54
	Sp1 SS/ss G2046T (rs1800012)	Husted (male)	2009	Whites	62	60.2±14.1	male	NA (male)	lumbar spine, femoral neck	osteoporosis	age, BMI	57	healthy	59.3±14.3	NA (male)	lumbar spine, femoral neck	yes	0.07	0.95	0.50	1.80	2.85	0.29	28.19	0.81	0.39	1.72	2.56	0.25	25.74	0.70	0.33	1.51
	Sp1 SS/ss G2046T (rs1800012)	Jin	2009	Whites	98	81.6±8.9	both	NA (male & female)	hip	low-trauma hip fracture		143	healthy	71.1±5.6	postmenopausal	NR	yes	0.08	1.01	0.63	1.62	1.48	0.36	6.06	0.96	0.56	1.66	1.44	0.35	5.96	0.90	0.51	1.58
	Sp1 SS/ss G2046T (rs1800012)	Tran	2009	Whites	332	71.5±7.3	female	NR	lumbar spine,femoral neck	fracture		583	non fracture	69.4±6.8	NR	lumbar spine, femoral neck	yes	0.21	1.20	0.95	1.52	2.87	1.41	5.85	1.09	0.82	1.45	2.85	1.39	5.84	0.92	0.69	1.23
	Sp1 SS/ss G2046T (rs1800012)	Falcon-Ramirez	2010	Mexicans	100	65.7±9.5	both	NR	lumbar spine	osteoporotic	race, gender	100	non osteoporotic	54.9±13	NR	lumbar spine	yes	0.09	4.36	2.56	7.40	11.58	0.63	212.21	6.58	3.52	12.32	24.32	1.31	451.96	5.32	2.86	9.88
	Sp1 SS/ss G2046T (rs1800012)	Erdogan	2010	Turkish	50	58.18±5.98	female	postmenopausal	lumbar spine, femoral neck	osteoporotic		30	normal bone mass	53.57 ±5.37	postmenopausal	lumbar spine, femoral neck	yes	0.05	1.02	0.47	2.21	0.58	0.08	4.37	1.15	0.45	2.93	0.63	0.08	4.88	1.31	0.50	3.47
	Sp1 SS/ss G2046T (rs1800012)	Ralston (Aarhus, female)	2006	Whites	247	NR	female	pre/post- menopausal	lumbar spine, femoral neck	fracture		341	no fractures		pre/post- menopausal	lumbar spine, femoral neck	NA														1.14	0.82	1.56
	Sp1 SS/ss G2046T (rs1800012)	Ralston (Aarhus, male)	2006	Whites	56	NR	male	NA (male)	lumbar spine, femoral neck	fracture		101	no fractures		NA (male)	lumbar spine, femoral neck	NA														0.98	0.86	1.18
	Sp1 SS/ss G2046T (rs1800012)	Ralston (Aberdeen, female)	2006	Whites	784	>18	female	pre/post- menopausal	lumbar spine, femoral neck	fracture		3102	no fractures		pre/post- menopausal	lumbar spine, femoral neck	NA														1.04	0.74	1.53
	Sp1 SS/ss G2046T (rs1800012)	Ralston (Amsterdam, female)	2006	Whites	391	>55	female	pre/post- menopausal	lumbar spine, femoral neck	fracture		394	no fractures		pre/post- menopausal	lumbar spine, femoral neck	NA														1.17	0.75	1.74
	Sp1 SS/ss G2046T (rs1800012)	Ralston (Amsterdam, male)	2006	Whites	327	>55	male	NA (male)	lumbar spine, femoral neck	fracture		401	no fractures		NA (male)	lumbar spine, femoral neck	NA														1.05	0.89	1.27
	Sp1 SS/ss G2046T (rs1800012)	Ralston (Barcelona, female)	2006	Whites	113	>45	female	pre/post- menopausal	lumbar spine, femoral neck	fracture		644	no fractures		pre/post- menopausal	lumbar spine, femoral neck	NA														1.15	0.89	1.47
	Sp1 SS/ss G2046T (rs1800012)	Ralston (EPOS, male)	2006	Whites	815	20- 80	male	pre/post- menopausal	lumbar spine, femoral neck	fracture		1698	no fractures		pre/post- menopausal	lumbar spine, femoral neck	NA														0.83	0.74	1.10
	Sp1 SS/ss G2046T (rs1800012)	Ralston (EPOS, female)	2006	Whites	407	20- 80	female	NA (male)	lumbar spine, femoral neck	fracture		1326	no fractures		NA (male)	lumbar spine, femoral neck	NA														0.79	0.58	1.36
	Sp1 SS/ss G2046T (rs1800012)	Ralston (Florence, female)	2006	Whites	270	NR	female	pre/post- menopausal	lumbar spine, femoral neck	fracture		1454	no fractures		pre/post- menopausal	lumbar spine, femoral neck	NA														1.09	0.93	1.28
	Sp1 SS/ss G2046T (rs1800012)	Ralston (Florence, male)	2006	Whites	76	NR	male	NA (male)	lumbar spine, femoral neck	fracture		555	no fractures		NA (male)	lumbar spine, femoral neck	NA														2.22	1.27	3.11
	Sp1 SS/ss G2046T (rs1800012)	Ralston (Graz, female)	2006	Whites	577	>21	female	pre/post- menopausal	lumbar spine, femoral neck	fracture		887	no fractures		pre/post- menopausal	lumbar spine, femoral neck	NA														1.13	0.79	1.61
	Sp1 SS/ss G2046T (rs1800012)	Ralston (Graz, male)	2006	Whites	172	>21	male	NA (male)	lumbar spine, femoral neck	fracture		243	no fractures		NA (male)	lumbar spine, femoral neck	NA														0.74	0.68	1.04
	Sp1 SS/ss G2046T (rs1800012)	Ralston (Oxagen, female)	2006	Whites	131	NR	female	pre/post- menopausal	lumbar spine, femoral neck	fracture		210	no fractures		pre/post- menopausal	lumbar spine, femoral neck	NA														1.17	0.77	1.72
	Sp1 SS/ss G2046T (rs1800012)	Ralston (Oxagen, male)	2006	Whites	104	NR	male	NA (male)	lumbar spine, femoral neck	fracture		117	no fractures		NA (male)	lumbar spine, femoral neck	NA														0.88	0.65	1.38
	Sp1 SS/ss G2046T (rs1800012)	Ralston (Rotterdam, female)	2006	Whites	1197	>55	female	postmenopausal	lumbar spine, femoral neck	fracture		3681	no fractures		99.7% postmenopausal	lumbar spine, femoral neck	NA														1.10	0.61	1.92
	Sp1 SS/ss G2046T (rs1800012)	Ralston (Rotterdam, male)	2006	Whites	400	>55	male	N/A (male)	lumbar spine, femoral neck	fracture		2705	no fractures		NA (male)	lumbar spine, femoral neck	NA														1.07	0.85	1.37
COL1A1	G-1997T (rs1107946)	Husted (female)	2009	Whites	228	65.1±8.2	female	pre/post- menopausal	lumbar spine, femoral neck	osteoporosis	age, BMI	226	healthy	65.3±8.2	pre/post- menopausal	lumbar spine, femoral neck	yes	0.11	1.44	0.99	2.08	1.40	0.44	4.48	1.52	1.00	2.31	1.55	0.48	4.98	1.49	0.97	2.31
	G-1997T (rs1107946)	Husted (male)	2009	Whites	63	60.2±14.1	male	NA (male)	lumbar spine, femoral neck	osteoporosis	age, BMI	57	healthy	59.3±14.3	NA (male)	lumbar spine, femoral neck	yes	0.06	1.27	0.64	2.53	0.90	0.06	14.78	1.38	0.63	2.99	1.00	0.06	16.53	1.40	0.64	3.08
	G-1997T (rs1107946)	Jin	2009	Whites	98	81.6±8.9	both	NA (male & female)	hip	low-trauma hip fracture		143	healthy	71.1±5.6	postmenopausal		yes	0.08	1.33	0.81	2.19	3.00	0.54	16.71	1.26	0.71	2.23	3.12	0.56	17.49	1.11	0.62	2.01
COL1A1	-1663indelT (rs2412298)	Husted female)	2009	Whites	227	65.1±8.2	both	pre/post- menopausal	lumbar spine, femoral neck	osteoporosis	age, BMI	225	healthy	65.3±8.2	pre/post- menopausal	lumbar spine, femoral neck	yes	0.12	1.13	0.81	1.59	1.59	0.60	4.17	1.09	0.74	1.62	1.60	0.61	4.24	1.01	0.67	1.52
	-1663indelT (rs2412298)	Husted (male)	2009	Whites	63	60.2±14.1	male	NA (male)	lumbar spine, femoral neck	osteoporosis	age, BMI	57	healthy	59.3±14.3	NA (male)	lumbar spine, femoral neck	yes	0.07	0.88	0.47	1.64	3.80	0.41	35.01	0.69	0.33	1.44	3.14	0.34	29.47	0.55	0.25	1.18
	-1663indelT (rs2412298)	Jin	2009	Whites	98	81.6±8.9	both	NA (male & female)	hip	low-trauma hip fracture		143	healthy	71.1±5.6	postmenopausal		yes	0.09	0.96	0.60	1.53	1.80	0.53	6.07	0.83	0.48	1.45	1.65	0.48	5.63	0.71	0.40	1.28
COL1A1	MspI 26kb upstream COL1A1 (mt-/wt+)	McGuigan	2000	Whites	93	70.2±5.6	both	NA (male & female)	lumbar spine	vertabral fracture	sge, sex	88	healthy	71.4±6.2	NA (male & female)	lumbar spine	yes	0.08	1.29	0.83	2.02	1.24	0.53	2.90	1.40	0.78	2.51	1.42	0.58	3.45	1.29	0.70	2.39
	MspI 26kb upstream COL1A1 (mt-/wt+)	Willing	1997	Whites	66	70.2	female	postmenopausal	femoral neck	low BMD	age	68	top quartile BMD	66.4	postmenopausal	femoral neck	yes	0.07	1.37	0.82	2.29	2.17	0.52	9.05	1.47	0.73	2.93	2.58	0.59	11.40	1.20	0.61	2.37
COL1A1	RsaI in intron 5 (mt-/wt+)	McGuigan	2000	Whites	93	70.2±5.6	both	NA (male & female)	lumbar spine	vertabral fracture	sge, sex	88	healthy	71.4±6.2	NA (male & female)	lumbar spine	yes	0.08	1.73	1.05	2.83	1.43	0.23	8.79	2.14	1.18	3.89	1.99	0.32	12.43	2.08	1.14	3.80
	RsaI in intron 5 (mt-/wt +)	Willing	1997	Whites	66	70.2	female	postmenopausal	femoral neck	low BMD	age	68	top quartile BMD	66.4	postmenopausal	femoral neck	yes	0.07	0.60	0.33	1.09	0.60	0.14	2.62	0.54	0.27	1.11	0.50	0.11	2.22	0.59	0.28	1.24
COL1A1	MnII in exon 52 (mt-/wt+)	McGuigan	2000	Whites	93	70.2±5.6	both	NA (male & female)	lumbar spine	vertabral fracture	sge, sex	88	healthy	71.4±6.2	NA (male & female)	lumbar spine	yes	0.10	0.68	0.44	1.06	0.37	0.15	0.89	0.82	0.45	1.46	0.38	0.15	0.96	1.34	0.73	2.44
	MnII in exon 52 (mt-/wt+)	Willing	1997	Whites	66	70.2	female	postmenopausal	femoral neck	low BMD	age	68	top quartile BMD	66.4	postmenopausal	femoral neck	yes	0.08	0.91	0.54	1.53	1.49	0.45	4.97	0.74	0.38	1.47	1.24	0.35	4.30	0.65	0.32	1.29
IGF-I	194-bp CA (mt+ /wt-)	Kim	2002	Whites	75	(45-75)	female	postmenopausal	lumbar spine& femoral neck	osteoporosis		67	normal	45-75	postmenopausal	lumbar spine, femoral neck	yes	0.07	0.76	0.43	1.35	0.06	0.00	1.13	0.99	0.51	1.96	0.07	0.00	1.26	1.48	0.74	2.98
IGF-I	wt=192bp CA	Rosen	1998	Whites	25	50.2±1.9	male	NA (male)	lumbar spine, hip	idiopathic osteoporosis		146	no osteoporosis	59.9	NA (male & female)	lumbar spine	NA								9	25	56	88					
IGF-I	wt=192bp CA	Lakatos	2004	Whites	22	60.8±8.8	female	pre/post- menopausal	lumbar spine, femoral neck	primary billiary cirrhosis & osteoporosis		48	pimary biliary cirrhosis w/o osteoporosis	56.2±11.1	pre/post- menopausal	lumbar spine, femoral neck	NA	0.17							16	22	30	48					
ACTN3	R577X	Delmonico	2008	Whites	1152	73.74±5.6	male	NA (male)	NR	osteoporosis		726	healthy	73.9±2.9	NA (male)	NR	yes	0.48	1.04	0.91	1.19	1.06	0.84	1.34	1.04	0.85	1.27	1.08	0.83	1.41	1.00	0.83	1.20
COL1A2	PvuII (mt-/wt+)	Willing	1997	Whites	66	70.2	female	postmenopausal	femoral neck	low BMD	age	68	top quartile BMD	66.4	postmenopausal	femoral neck	yes	0.08	1.69	1.03	2.78	1.61	0.64	4.06	2.17	1.07	4.40	2.38	0.86	6.58	1.62	0.81	3.21
COL1A2	RsaI (mt-/wt+)	Willing	1997	Whites	66	70.2	female	postmenopausal	femoral neck	low BMD	age	68	top quartile BMD	66.4	postmenopausal	femoral neck	yes	0.07	1.05	0.64	1.72	0.71	0.27	1.91	1.31	0.65	2.63	0.89	0.31	2.57	1.52	0.77	3.00
IGF-IR	G3174A	Lee	2008	East Asian	286	56.47±1.5 (43-76)	female	postmenopausal	lumbar spine, femoral neck, trochanter, Ward's triangle	osteoporosis & osteopenia		81	normal	56.47±1.5 (43-76)	postmenopausal	lumbar spine, femoral neck, trochanter, Ward's triangle	yes	0.13	0.60	0.42	0.87	0.48	0.24	0.97	0.54	0.32	0.90	0.36	0.17	0.78	0.76	0.46	1.25
ITGB4	176T>C Leu33Pro (rs5920)	Tofteng (female)	2007	Whites	174	47 (38-55)	female	pre/post- menopausal	lumbar spine, hip	hip fracture		5104	no hip fracture	47 (38-55)	pre/post- menopausal	lumbar spine, hip	yes	0.13	1.04	0.79	1.39	1.75	0.84	3.64	0.97	0.69	1.35	1.70	0.81	3.55	0.87	0.61	1.24
	176T>C Leu33Pro (rs5920)	Tofteng (male)	2007	Whites	93	47 (38-55)	male	NA (male)	lumbar spine, hip	hip fracture		4129	no hip fracture	47 (38-55)	NA (male)	lumbar spine, hip	yes	0.07	1.50	1.06	2.13	2.11	0.84	5.29	1.55	1.02	2.36	2.38	0.94	6.07	1.40	0.91	2.16

NR: not reported
NA: not applicable
