# Supplementary material for: Synopsis and meta-analysis of genetic association studies in osteoporosis for the focal adhesion family genes: the CUMAGAS-OSTEOporosis information system
Source: BMC Med. 2011 Jan 26;9:9. doi: 10.1186/1741-7015-9-9 (PMC3040157; doi:10.1186/1741-7015-9-9)
Supplement: Additional file 3 — Supplementary Figure 1 [file 1741-7015-9-9-S3.PDF]

Supplementary figure 1. Random effects (RE) odds ratio (OR) estimates with the corresponding 95% confidence interval (CI) for the overall analysis for the dominant model of COL1A1 2046T allele. The OR estimate of each study is marked with a solid black square. The size of the square represents the weight that the corresponding study exerts in the meta-analysis. The arrow indicates a limit to zero. The confidence intervals of pooled estimates are displayed as a horizontal line through the diamond; this line might be contained within the diamond if the confidence interval is narrow. The horizontal axis is plotted on a log scale.

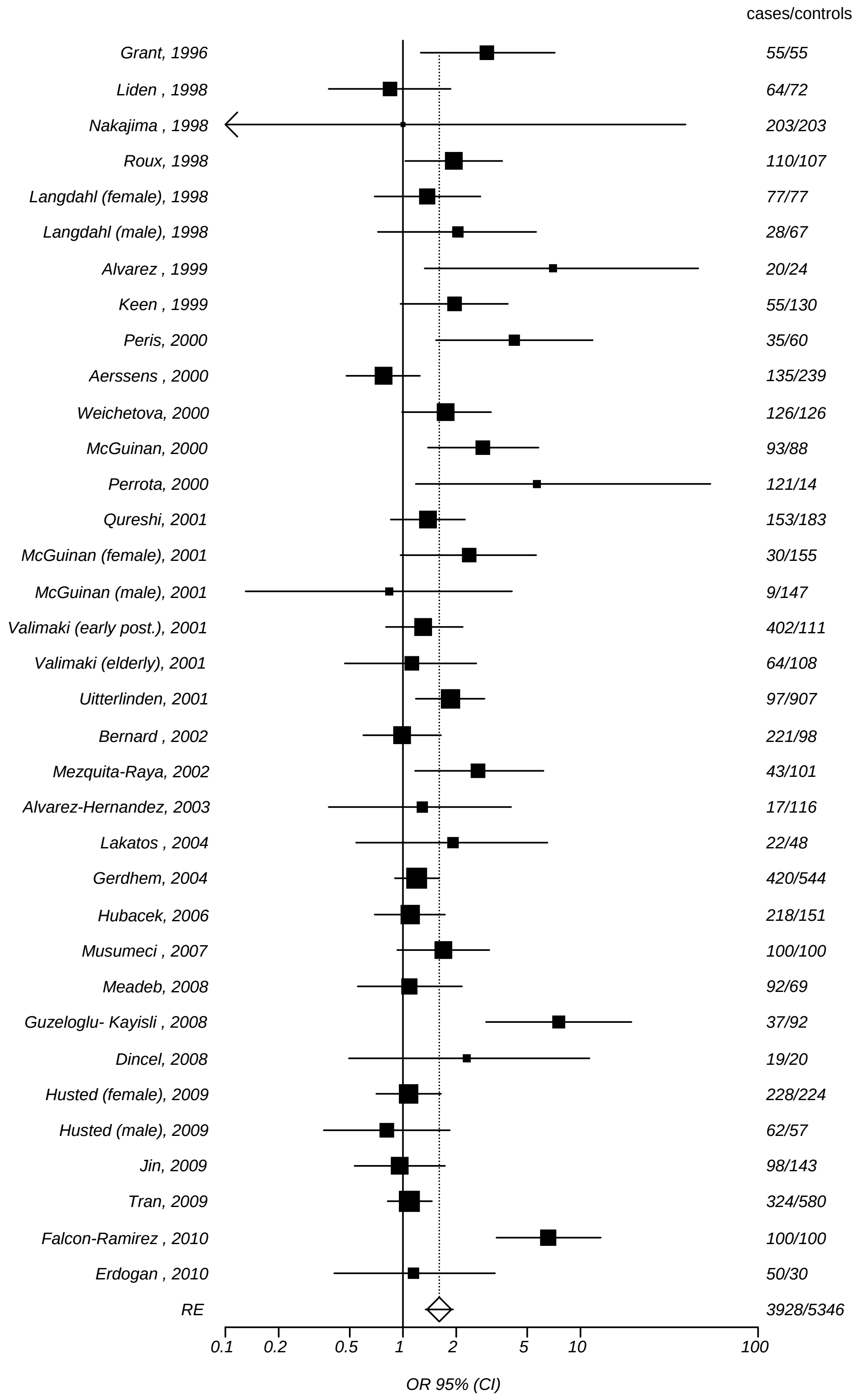
cases/controls
Grant, 1996 55/55
Liden , 1998 64/72
Nakajima , 1998 203/203
Roux, 1998 110/107
Langdahl (female), 1998 77/77
Langdahl (male), 1998 28/67
Alvarez , 1999 20/24
Keen , 1999 55/130
Peris, 2000 35/60
Aerssens , 2000 135/239
Weichetova, 2000 126/126
McGuinan, 2000 93/88
Perrota, 2000 121/14
Qureshi, 2001 153/183
McGuinan (female), 2001 30/155
McGuinan (male), 2001 9/147
Valimaki (early post.), 2001 402/111
Valimaki (elderly), 2001 64/108
Uitterlinden, 2001 97/907
Bernard , 2002 221/98
Mezquita-Raya, 2002 43/101
Alvarez-Hernandez, 2003 17/116
Lakatos , 2004 22/48
Gerdhem, 2004 420/544
Hubacek, 2006 218/151
Musumeci , 2007 100/100
Meadeb, 2008 92/69
Guzeloglu- Kayisli , 2008 37/92
Dincel, 2008 19/20
Husted (female), 2009 228/224
Husted (male), 2009 62/57
Jin, 2009 98/143
Tran, 2009 324/580
Falcon-Ramirez , 2010 100/100
Erdogan , 2010 50/30
RE 3928/5346
0.1 0.2 0.5 1 2 5 10 100
OR 95% (CI)
